# Identified Optimal Codons and Phylogenetic Relationship in *Pseudobagrus* Species Based on Complete Mitogenomes

**DOI:** 10.3390/ani16020279

**Published:** 2026-01-16

**Authors:** Qian Qi, Weixu Jiang, Yanhui Sun, Feng Yang, Chenran Lv, Xiaolong Gao, Liming Zhao, Gao Gao, Cheng Zhang

**Affiliations:** 1 Henan Open Laboratory of Key Subjects of Environmental and Animal Products Safety, College of Animal Science and Technology, Henan University of Science and Technology, Luoyang 471023, China; qiqian@haust.edu.cn (Q.Q.);; 2 Animal Disease Prevention and Control Center of Jiyuan Industry City Integration Demonstration Zone, Jiyuan 459000, China; 3 Henan Province Aquatic Products Technology Extension Station, Zhengzhou 450003, China; 4 College of Animal Science and Technology, Yunnan Agricultural University, Kunming 650201, China

**Keywords:** *Pseudobagrus*, mitogenome, phylogenetic relationship, evolution, optimal codons

## Abstract

The genus *Pseudobagrus* represents a group of economically and ecologically significant freshwater bagrid catfishes in East Asia, yet its taxonomy remains contentious. This study employed the complete mitochondrial genomes of 15 *Pseudobagrus* species to clarify their phylogenetic relationships. The mitogenomes ranged from 16,526 to 16,647 bp, exhibiting a conserved gene order and significant AT bias (average A + T = 57.85%). All genomes contained 13 protein-coding genes (PCGs), 22 tRNAs, two rRNAs, and a control region. Start codons were predominantly ATG, except for *COI* (GTG), while stop codons varied among TAA, TAG, and incomplete T--. Codon usage bias favored NNU and NNA codons, and 12 optimal codons were identified in *P. albomarginatus*. The phylogenetic trees based on concatenated PCGs revealed two major clades. Clade I contained 14 species. Within this clade, *P. albomarginatus*, *P. tenuis*, and *P. brevicorpus* clustered together first, and then this trio grouped with *P. ussuriensis*. *Pseudobagrus trilineatus* formed the separate Clade II. These results provide a molecular foundation for species delimitation and systematic revision within *Pseudobagrus*, supporting the monophyly of the genus while highlighting cryptic diversity and taxonomic complexity.

## Simple Summary

The classification of *Pseudobagrus*, a group of valuable East Asian freshwater fishes, has long been unclear. This study aimed to clarify their evolutionary relationships and identify optimal genetic codes by analyzing the complete mitochondrial genes of 15 species. Results showed these genes have a stable structure with more A/T bases, and 12–15 optimal genetic codes were found in different species. The fishes fell into two main evolutionary groups, supporting the genus’ unity while revealing hidden diversity. These findings provide a genetic basis for accurate species identification and classification revision, which will aid sustainable aquaculture development and aquatic ecosystem protection.

## 1. Introduction

The genus *Pseudobagrus* comprises a group of freshwater benthic fishes within the family Bagridae (order Siluriformes), primarily distributed across East Asia. Their range encompasses river systems and streams in northern Vietnam, China (including Taiwan and Hainan Island), the Korean Peninsula, Japan, and the Far East region of Russia [1]. Species of this genus have tender flesh, high nutritional value, and recognized medicinal properties, affording them significant potential for economic development [2,3,4]. As integral components of freshwater ecosystems, their characteristics—such as habitat preferences, trophic level, and population dynamics—are crucial for understanding the structure and function of aquatic ecosystems. Within the framework of the “One Health” concept, comprehending the interrelationships between the health status of these aquatic organisms, environmental factors, and human activities holds particular value for achieving the green and sustainable development of aquaculture.

However, the taxonomic system of *Pseudobagrus* has long been contentious. Since Bleeker (1858) established the genus with *Bagrus aurantiacus* (Temminck & Schlegel, 1846) as the type species, the generic-level classification of Bagridae fishes in East Asia has undergone multiple revisions [5]. Ng and Freyhof proposed merging the traditionally defined genera *Tachysurus* and *Leiocassis* into *Pseudobagrus* [6]. Furthermore, molecular phylogenetic studies by Hardman, Ku, and colleagues have indicated that East Asian bagrid fishes form a monophyletic group. However, their work also revealed that the traditional genera *Pseudobagrus*, *Tachysurus*, and *Leiocassis* are each para- or polyphyletic, supporting a systematic revision and merging of these groups [7,8]. Based on mitochondrial *COI* gene analysis, Zou found that the monophyletic nature of genera *Pelteobagrus*, *Pseudobagrus* and *Leiocassis* did not exist [9]. With the advancing application of molecular phylogenetics and integrative taxonomy, significant progress has been made in delineating *Pseudobagrus* species and discovering new taxa. For instance, *Pseudobagrus* specimens collected from the Bazhi River in Hekou, Yunnan, were identified as *P. vietnamensis*, a species newly recorded in China. Researchers Cheng et al. reclassified populations originally identified as *P. albomarginatus* within the Pearl River basin as a new species, *Tachysurus lani*, consequently restricting the confirmed distribution of true *P. albomarginatus* to the lower Yangtze River, Qiantang River, and Huai River basins [10]. In an integrative study of the *P. albomarginatus* species complex, Cheng and Nichols not only reinstated the taxonomic status of *P. analis* and *P. similis* but also revealed the presence of morphologically cryptic yet genetically distinct lineages within *P. zhangfei* [10,11]. This suggests the potential existence of different Evolutionarily Significant Units (ESUs) within this taxon, necessitating attention in species conservation and management. Despite these efforts, a clearly defined and stable species classification for the genus *Pseudobagrus* remains elusive. The mitochondrial genome is an extranuclear genetic element capable of independent replication, transcription, and translation [12,13]. Owing to its relatively simple molecular structure, strict maternal inheritance, and rapid evolutionary rate, the mitochondrial genome has been widely employed in studies of fish population genetics, phylogenetics, and biogeography [14,15,16].

Therefore, this study aims to sequence the complete mitochondrial genomes of *Pseudobagrus* species and conduct comparative genomic and phylogenetic analyses. The primary objective is to reconstruct species delimitation within the genus at the molecular level. This work is intended to provide a genetic basis for understanding the adaptive potential of this group in changing environments.

## 2. Materials and Methods

### 2.1. Sample Collection and DNA Extraction

From May to June 2024, 176 specimens of *Pseudobagrus ussuriensis* were collected from the Luo River Basin in Luoning County, Luoyang City, Henan Province, China, and were farmed in the Breeding Base of Luoyang Daqianping Fishery Co., Ltd., Luoyang City, Henan Province, China. A sample of the *P. ussuriensis* was randomly collected and injected with MS222, the fish was aseptically dissected, and the pectoral fins were excised and preserved in absolute ethanol. Genomic DNA was extracted from approximately 100 mg of pectoral fin tissue using a E.Z.N.A.^®^ Tissue DNA Kit (OMEGA, Norwalk, CT, USA), following the manufacturer’s protocol. The purity and concentration of the extracted DNA were assessed by 1% agarose gel electrophoresis and spectrophotometry using a NanoDrop instrument (Thermo Fisher Scientific, Waltham, MA, USA). This high-quality DNA sample was stored in the Freshwater Fish Genetics and Breeding Laboratory of the School of Animal Science and Technology, Henan University of Science and Technology, with the serial number OQ156515123.

### 2.2. Sequencing

Sequencing was outsourced to Sangon Biotech (Shanghai) Co., Ltd. (Shanghai, China). The procedure was as follows: Sequencing libraries were constructed from the extracted DNA samples using the TruSeq^®^ Nano DNA Sample Prep Kit (Illumina, San Diego, CA, USA). The DNA was fragmented into segments of approximately 300–500 bp using a Covaris M220 focused-ultrasonicator (Woburn, MA, USA), and adapters were ligated to both ends. Bridge PCR amplification was subsequently performed. Paired-end sequencing of the sample DNA was conducted using the Illumina NovaSeq 6000 platform (San Diego, CA, USA). To ensure the accuracy of subsequent assembly, the raw sequencing data were subjected to quality control and filtering with the following steps: adapter sequences were removed from the reads; nucleotides other than A, G, C, or T at the 5′ end were trimmed; low-quality bases (with a quality score below Q20) at the ends of reads were trimmed; reads containing more than 10% of undetermined bases (N) were discarded; and finally, fragments shorter than 75 bp after adapter removal and quality trimming were excluded. The raw data obtained amounted to 5040.4 Mb. After quality control, 4925.9 Mb of clean data were retained, yielding an average sequencing depth of 294.45×.

### 2.3. Complete Mitochondrial Genome Assembly and Analysis

Clean reads were assembled to obtain the complete mitochondrial genome sequence using SPAdes v3.14.1 (https://github.com/ablab/spades, accessed on 6 January 2026), involving steps of error correction, alignment, and contig assembly [17]. The complete mitochondrial genome map was visualized using the online tool OrganellarGenomeDRAW version 1.3.12 [18]. Tandem repeats within the control region were identified using Tandem Repeats Finder program version 4.09 [19]. Furthermore, transfer RNA (tRNA) genes were identified, and their characteristic cloverleaf secondary structures and anticodons were verified using the online server tRNAscan-SE 2.03 [20]. Codon usage patterns for protein-coding genes (PCGs) and the nucleotide composition of the mitochondrial genome were determined using MEGA software version 5.0 [21].

### 2.4. Identification of Optimal Codons

Various indices related to codon usage bias were calculated using the online program EMBOSS explorer (http://www.bioinformatics.nl/emboss-explorer/, accessed on 6 January 2026). These indices included the GC content at the first (GC1), second (GC2), and third (GC3) codon positions, the overall GC content (GCall), the Codon Adaptation Index (CAI), and the Effective Number of Codons (ENC) [22,23]. The Relative Synonymous Codon Usage (RSCU) values were computed using CodonW v1.4.2 [24]. Optimal codons are defined as those over-represented in highly expressed genes compared to lowly expressed genes [25]. In this study, genes were ranked based on their ENC values. The top 10% and bottom 10% of genes, possessing the lowest and highest ENC values, respectively, were designated as the high-expression and low-expression datasets. A codon was considered optimal if its RSCU value was greater than 1 in the high-expression group, less than 1 in the low-expression group, and the absolute difference in RSCU values between the two groups (|ΔRSCU|) was greater than 0.08.

### 2.5. Phylogenetic Analysis

To investigate phylogenetic relationships, the complete mitochondrial genomes of selected species from the genus *Pseudobagrus* were utilized. Among them, the mitogenome sequences of *P. tokiensis* (AB054127.1), *P. brevicorpus* (NC_015625.1), *P. brevicaudatus* (NC_021393.1), *P. truncatus* (NC_021395.1), *P. trilineatus* (NC_022705.1), *P. ondon* (NC_022725.1), *P. albomarginatus* (NC_022726.1), *P. emarginatus* (NC_024279.1), *P. koreanus* (NC_028434.1), *P. tenuis* (NC_035498.1), *P. medianalis* (NC_037048.1), *P. pratti* (NC_041443.1), *P. brachyrhabdion* (NC_064133.1), and *P. gracilis* (NC_068707.1) were all from NCBI. *Hemibagrus punctatus* (NC_087772.1) was selected as the outgroup. The nucleotide sequences of the 13 PCGs from these species were aligned using MAFFT v7 with default parameters and subsequently concatenated into a single supermatrix. The optimal nucleotide substitution model for Bayesian Inference (BI) was determined to be GTR + I + G using jModelTest v2, based on the Bayesian Information Criterion (BIC) [26]. The BI analysis was performed using MrBayes 3.2.7a, running four simultaneous Markov Chain Monte Carlo (MCMC) chains for 200,000 generations. The first 25% of generations were discarded as ‘burn-in’, and the remaining trees were used to construct a consensus topology and calculate Bayesian Posterior Probabilities [27]. Additionally, we constructed the maximum likelihood (ML) tree using RAxML (version 8) [28], with the GTR + GAMMA model identified as the optimal evolutionary framework, and the number of the bootstrap replicates was 1000. The resulting phylogenetic tree was visualized using FigTree v1.4.4.

## 3. Results

### 3.1. Composition and Structure of the Mitochondrial Genomes

The mitochondrial genome sequences of the 15 *Pseudobagrus* species were found to range in length from 16,526 to 16,647 bp, with the shortest being *P. brevicorpus* and the longest being *P. medianalis*. The average contents of bases A, T, G, and C in these mitochondrial genomes were 31.33%, 26.51%, 15.13%, to 27.03%, respectively. The A + T content (57.85%) was significantly higher than the C + G content (42.15%), indicating a distinct AT bias. Furthermore, the average AT-skew [(A − T)/(A + T)] was 0.08, and the average GC-skew [(G − C)/(G + C)] was −0.28. All species exhibited positive AT-skew values and negative GC-skew values, suggesting a strong bias towards adenine and against cytosine in the mitochondrial genome composition (Table 1). The mitochondrial genomes of all *Pseudobagrus* species were identified as circular, double-stranded molecules with a consistent gene order, comprising 13 protein-coding genes (PCGs), 2 ribosomal RNA (rRNA) genes, 22 transfer RNA (tRNA) genes, and one major non-coding control region (D-loop). With the exception of the *ND6* gene and eight tRNA genes (tRNA-Gln, Ala, Asn, Cys, Tyr, Ser (UGA), Glu, and Pro) located on the light strand (L-strand), the remaining genes were encoded on the heavy strand (H-strand) (Figure S1).

### 3.2. Transfer RNAs

All tRNA genes were successfully identified using the tRNAscan-SE 2.0 tool. The mitochondrial genomes of the 15 *Pseudobagrus* species each contained 22 tRNA genes, which were distributed across different regions of the genome and ranged in length from 67 to 75 bp. Specifically, 14 tRNA genes were encoded on the H-strand, while the remaining 8 were located on the L-strand. The ancestral arrangement order of these tRNA genes was conserved and consistent with the pattern commonly observed in vertebrates. Two serine tRNAs (tRNA-Ser (UCN) and tRNA-Ser (AGY)) and two leucine tRNAs (tRNA-Leu (UUR) and tRNA-Leu (CUN)) were identified. A unique feature was observed in *P. medianalis*, where the entire gene sequence for tRNA-Val was located within the 16S rRNA gene sequence; this phenomenon was not found in the other 14 species. In the 15 *Pseudobagrus* species, with the exception of *P. koreanus* whose *trnS1* lacked the dihydrouridine (D) arm, the remaining 22 tRNAs could be folded into the typical cloverleaf secondary structure. The nucleotide composition of these 22 tRNAs was highly conserved among the *Pseudobagrus* species, indicating considerable stability compared to other genes in the mitochondrial genome. Notably, the anticodon loops of tRNA-Thr and tRNA-Val were extended to 9 bp, compared to the typical 7 bp. Furthermore, non-canonical base pairs (e.g., G-U wobble pairs) or mismatches were frequently observed in the otherwise conserved tRNA structures. Among the species studied, the lowest number of G-U base pairs (22) was identified in *P. brachyrhabdion*. A count of 26 pairs was recorded for *P. albomarginatus* and *P. brevicorpus*, while 27 pairs were found in *P. koreanus* and *P. pratti*. The value of 28 pairs was shared by four species: *P. brevicaudatus*, *P. medianalis*, *P. truncatus*, and *P. tokiensis*. A total of 29 pairs were observed in *P. trilineatus*, *P. ondon*, and *P. ussuriensis*, and 30 pairs were detected in *P. emarginatus* and *P. tenuis*. The highest number of G-U base pairs (31) was observed in *P. gracilis*. Given that G-U pairing forms a weak bond and represents a non-canonical base pair in tRNA secondary structures, it is postulated that G-U pairing might be a common phenomenon in mitochondrial tRNA genomes and could potentially be corrected via post-transcriptional editing mechanisms. Additionally, as mitochondrial genomes are not subject to recombination processes, such base mismatches might contribute to the elimination of deleterious mutations (Figure S2).

### 3.3. Ribosomal RNAs

With the exception of *P. medianalis* (whose tRNA-Val sequence was embedded within the *16S* rRNA), the mitochondrial genomes of the other 14 *Pseudobagrus* species contained two rRNA subunits. The *12S* rRNA and *16S* rRNA genes, separated by tRNA-Val, were located on the H-strand. The total length of the two rRNA subunits varied among the 15 species, ranging from 2610 to 2704 bp. The A + T content of these rRNAs ranged between 56.56% and 58.24%, slightly higher in 16S rRNA than in 12S rRNA. Both rRNA genes exhibited negative GC-skew and positive AT-skew values, indicating a higher content of adenine and cytosine bases within these genes. All 15 species possessed one origin of light-strand replication (*OL*) and one *D-loop* region. The *D-loop* was the longest non-coding region, located between tRNA-Pro and tRNA-Phe, with a length ranging from 890 to 893 bp. The *OL* was the second longest non-coding region, situated downstream of tRNA-Asn and upstream of tRNA-Cys. This region could be folded into a stable stem-loop secondary structure, featuring an 18 bp stem and a loop of either 12 or 15 bp (Table S1).

### 3.4. Protein-Coding Genes

**Figure 1:**
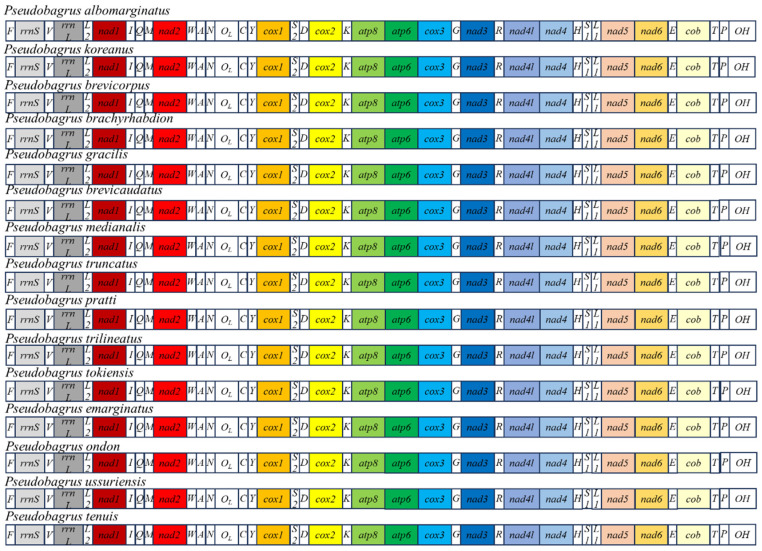
Gene arrangement of mitochondrial genomes from the 15 *Pseudobagrus*.

The combined length of the 13 PCGs in the 15 *Pseudobagrus* species ranged from 11,406 to 11,412 bp, encoding 3802 to 3804 amino acids. Among these 13 PCGs, only the *ND6* gene was encoded on the L-strand, while the other 12 were located on the H-strand. The *COI* gene was initiated by a GTG codon, whereas the other PCGs used the traditional ATG start codon; this feature is not unique to *Pseudobagrus* but has also been observed in other teleost fishes. Three types of termination codons were identified: two complete stop codons (TAA, TAG) and one incomplete stop codon (T--). Their usage frequency, from highest to lowest, was TAA, T--, and TAG. The *ATP8*, *COIII*, *ND4L*, *ND5*, *Cyt b*, and *ATP6* genes consistently used the typical TAA stop codon. However, the termination codons for the remaining 6 PCGs varied among species. For instance, in *P. emarginatus*, the *ND2* and *ND3* genes were terminated by TAG, whereas in the other 14 species, they were terminated by an incomplete stop codon T-- (Table S1). The *ND1* gene termination codon was TAA in *P. koreanus*, *P. medianalis*, *P. truncatus*, *P. pratti*, *P. trilineatus*, and *P. emarginatus*, but was TAG in the other 9 species. The variability of stop codons in fish mitochondrial genomes suggests a potential for alteration, possibly indicative of a rapid evolutionary process. It is hypothesized that the incomplete stop codons are completed via post-transcriptional polyadenylation, a common mechanism in metazoan mitochondrial genomes. The base composition of the 13 PCGs varied among the 15 species, with base A being the most abundant and base G the least. The A + T content of the PCGs ranged from 56.56% to 58.78%, dominated by thymine and adenine nucleotides. The average lengths of the 13 PCGs were calculated for the 15 species. The *ATP8* gene was found to be the shortest with an average length of 168 bp, whereas *ND5* was the longest with an average length of 1827 bp. Furthermore, the *COI* gene length was conserved at 1551 bp across all 15 species: *P. brevicorpus*, *P. ussuriensis*, *P. brevicaudatus*, *P. truncatus*, *P. trilineatus*, *P. ondon*, *P. albomarginatus*, *P. emarginatus*, *P. koreanus*, *P. tenuis*, *P. medianalis*, *P. pratti*, *P. brachyrhabdion*, *P. gracilis*, and *P. tokiensis*. Analysis of AT-skew and GC-skew values for all PCGs revealed that the *COI*, *ND3*, *ND4L*, and *ND6* genes consistently exhibited positive AT-skew values across all 15 species. With the exception of the *ND6* gene, all other PCGs showed negative GC-skew values. Additionally, the gene arrangement of the 15 *Pseudobagrus* species was the same as that of most teleost fishes (Figure 1). These results showed that the gene arrangement of the *Pseudobagrus* species was conservative.

### 3.5. Selection of Optimal Codons

The GC content and Effective Number of Codons (ENC) values for the 13 PCGs were analyzed in *P. albomarginatus*. The values for GC, GC1, GC2, GC3, and ENC were 0.430, 50.51%, 40.58%, 31.68%, and 55.950, respectively. Similar trends were observed in the other 14 *Pseudobagrus* species (Table 2). Furthermore, all 15 investigated species exhibited the lowest GC content at the third codon position (GC3) and the highest at the first position (GC1) for the 13 PCGs, indicating a general pattern of GC content across codon positions: GC1 > GC2 > GC3. Specifically, the *nad2* and *ATP8* genes showed the lowest GC2 and highest GC1 contents among the *Pseudobagrus* species. Pearson correlation analysis revealed significant correlations between overall GC content (GCall) and GC1, GC2, and GC3, but not with other parameters. The Relative Synonymous Codon Usage (RSCU) values were calculated to assess codon usage patterns. The average frequency of codons for all PCGs across the 15 species is presented in Table S2. RSCU is a direct indicator of codon usage bias. A strong bias was observed, with RSCU values for NNU and NNA codons mostly greater than 1, indicating their relatively high usage frequency. The RSCU pattern demonstrated a preference for codons ending with adenine (A) at the third codon position across various amino acid synonymous substitutions. Using *P. albomarginatus* as an example, based on ENC values, the *ND3* gene (highest ENC) and the *ND6* gene (lowest ENC) were identified as representing high and low expression groups, respectively. Using the ‘1 RSCU’ method, 12 optimal codons were determined: CUA, GUA, UCC, CCC, ACU, ACA, GCC, CAA, GAA, UGC, CGC, and GGC (Tables S3 and S4). Applying the same criteria, 9 optimal codons (CUU, CUA, AUU, ACA, GCC, AAC, AAA, GAA, UGG) were identified for *P. koreanus*, and 15 (CUU, AUU, GUA, UCU, UCC, CCU, ACU, ACA, GCC, UAU, CAU, AAA, GAU, GAA, UGC) for *P. brevicorpus*. Numerous studies suggest that Codon Usage Bias (CUB) is associated with multiple factors and can be determined either by mutation pressure or by a combination of natural selection and mutation. Generally, natural selection influencing translational efficiency and directional mutation pressure on DNA sequences are two key factors explaining CUB variations among species and within genomes. The results of this study indicate that, apart from natural selection, mutation pressure might not significantly affect CUB in these species.

### 3.6. Phylogenetic Analysis

**Figure 2:**
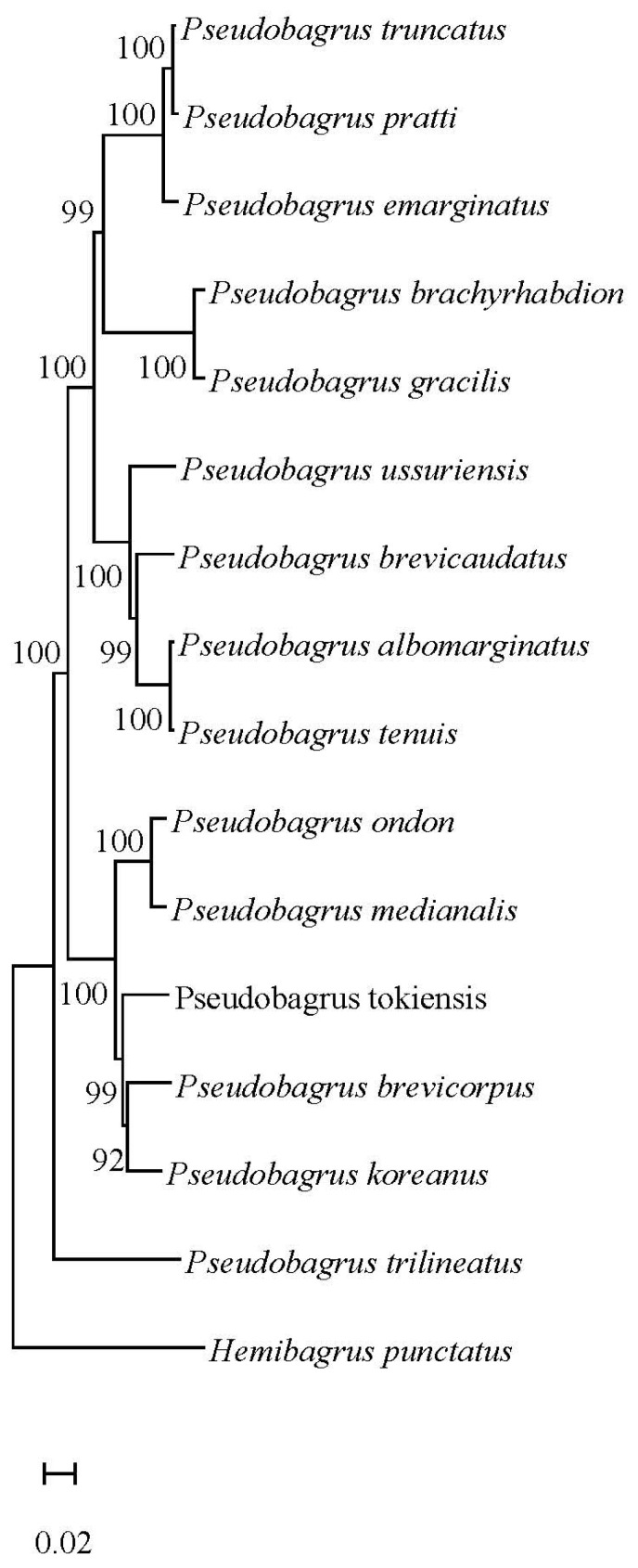
A maximum likelihood tree for *Pseudobagrus* species constructed based on 13 concatenated protein coding gene sequences. *Hemibagrus punctatus* was used as an outgroup. Numbers at each branch indicate maximum likelihood (ML) bootstrap values (%). The meaning of the scale bar is the unit length of the difference value between organisms or sequences.

**Figure 3:**
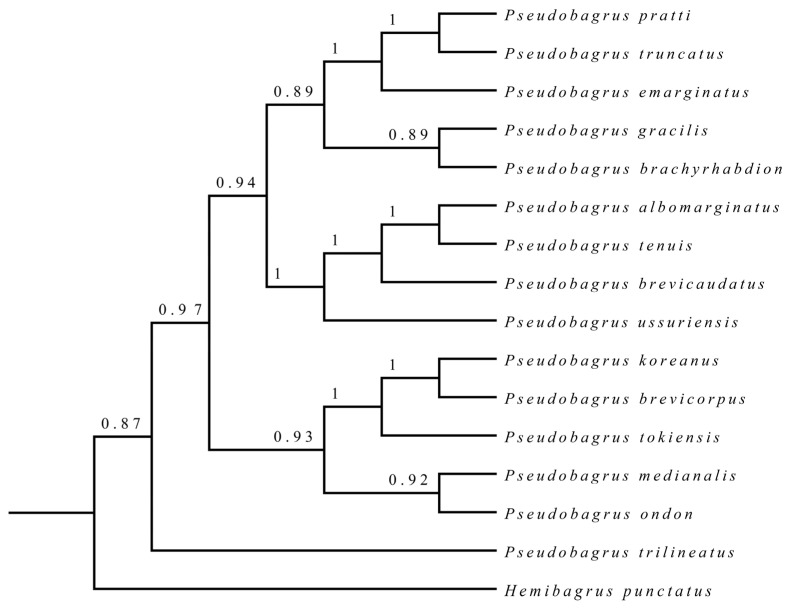
Phylogenetic tree of *Pseudobagrus* based on the Bayesian analysis of 13 concatenated protein coding gene sequences. *Hemibagrus punctatus* was used as an outgroup. Numbers at each branch indicate Bayesian posterior probabilities.

Bayesian inference (BI) and maximum likelihood trees were constructed using the concatenated sequences of the mitochondrial 13 protein-coding genes (PCGs) from the 15 *Pseudobagrus* species. Both ML bootstrap and Bayesian posterior probability values were high, and the topological structure of the two trees were consistent. The results all revealed that the 15 examined *Pseudobagrus* species were divided into two primary clades. Clade I comprised 14 species, while *P. trilineatus* formed Clade II. Within Clade I, a specific topology was recovered. Both *P. albomarginatus* and *P. tenuis* were initially clustered together, demonstrating a close phylogenetic relationship. This group subsequently clustered with *P. brevicorpus*. These three species then formed a cluster with *P. ussuriensis*. Similarly, *P. truncatus* and *P. pratti* were closely related and clustered together first. This pair then clustered with *P. emarginatus* to form another clade. Finally, these four species clustered with *P. gracilis* and *P. brachyrhabdion*, forming a distinct clade. In a separate grouping, *P. brevicorpus* and *P. koreanus* were initially clustered, which was then joined by *P. tokiensis*. These three species ultimately clustered with *P. ondon* and *P. medianalis* forming another clade (Figure 2 and Figure 3).

## 4. Discussion

The mitochondrial genomes of the 15 *Pseudobagrus* species analyzed in this study all exhibited the typical circular, double-stranded molecular structure. Their lengths ranged from 16,526 bp (*P. brevicorpus*) to 16,647 bp (*P. medianalis*), consistent with the length range observed in the vast majority of teleost fishes [12]. Gene composition and arrangement were highly conserved, with all genomes containing 13 protein-coding genes (PCGs), 22 tRNA genes, 2 rRNA genes, and one major non-coding control region (*D-loop*). The gene order was identical to the putative ancestral arrangement for vertebrates, with no large-scale gene rearrangements detected [12,13]. This high degree of structural conservation implies strong functional constraints on the mitochondrial genome to maintain its core function in cellular energy metabolism [29]. Regarding nucleotide composition, all species exhibited a significant AT bias, with an average A + T content of 57.85% (range: 56.56–58.78%). This characteristic aligns with findings from numerous other teleost mitochondrial genomic studies; for instance, *Osteochilus salsburyi* exhibits an A + T content of 59.61% [30], while 11 species within the genus *Pseudogastromyzon* show a range of 54.10–55.03% [31]. This pervasive AT bias is likely attributable to the combined effects of mutational pressure, strand asymmetry during replication, and natural selection [14]. Further analysis of strand-specific nucleotide bias revealed positive AT-skew and negative GC-skew values across all species, indicating an enrichment of adenine and a depletion of guanine on the heavy strand (H-strand). This bias (A-skew and C-skew) is a typical feature of vertebrate mitochondrial genomes and is thought to result from the prolonged single-stranded state of the H-strand during replication, making it more susceptible to cytosine deamination into uracil [32]. Notably, *P. medianalis* possessed the largest genome, primarily due to an elongated control region. As the most rapidly evolving region of the mitochondrial genome, length variation in the control region is often caused by insertions or deletions of tandem repeat sequences. Similar control region length polymorphisms have been reported, for example, in *Pseudogastromyzon changtingensis tungpeiensis*, whose control region is significantly longer than that of its close relative *P. changtingensis changtingensis* [31]. Such length variations in non-coding regions are generally considered the result of species-specific evolutionary events. While they may not convey strong phylogenetic signals, they provide potential molecular markers for population genetic studies.

In-depth analysis of the 13 PCGs revealed their evolutionary patterns under varying functional constraints. Start codon usage was highly conserved: with the exception of the *COI* gene, which used GTG, the other 12 PCGs universally employed the canonical ATG start codon. The use of GTG as the start codon for *COI* is extremely prevalent in teleosts, as explicitly reported in *O. salsburyi* [30], all species of the genus *Pseudogastromyzon* [31], and *Cheilopogon doederleinii* [33]. This suggests that GTG as the start codon for *COI* was likely fixed early in fish mitochondrial evolution and may be functionally ensured through an unknown translation initiation mechanism or synergy with specific flanking sequences. In contrast to the conservation of start codons, stop codon usage demonstrated considerable variability. We identified three types of stop codons: the canonical TAA and TAG, and incomplete T-- or TA-. Incomplete stop codons were frequently observed in genes such as *ND2*, *COII*, and *Cyt b*. It is widely accepted that these incomplete stop codons (typically T or TA) are completed to a UAA stop codon via post-transcriptional polyadenylation, a widespread and economical transcription termination mechanism in metazoan mitochondrial genomes [34]. Notably, the usage of certain stop codons exhibited species-specificity. In *P. emarginatus*, the *ND2* and *ND3* genes terminated with TAG, whereas they were terminated by an incomplete T-- codon in the other 14 species. This “plasticity” or “rapid evolution” of stop codons has been observed in mitochondrial genomes of various fish species, such as *Gymnogobius petschiliensis* [35] and *Cranoglanis bouderius* [36], suggesting they may be under weaker purifying selection or involved in fine-tuning specific gene expression regulation.

All 22 tRNA genes were identified by tRNAscan-SE software, with total lengths ranging from 1559 to 1560 bp. With the exception of the *trnS1*(GCT) gene in *P. koreanus*, which lacked the dihydrouridine (DHU) arm and thus could not form the canonical cloverleaf secondary structure, all other tRNAs were predicted to fold into the classic cloverleaf structure. The lack of a DHU arm in *trnS1*(GCT) is not uncommon in animal mitochondrial tRNAs; for instance, it has also been observed in the tRNA-Cys of *O. melanopleurus* [30]. This structural simplification is believed to be functionally compensated through interactions with other RNAs or protein factors and represents a trend in mitochondrial tRNA evolution. This study also found that the anticodon loops of *trnT* and *trnV* were extended to 9 bp instead of the typical 7 bp. This feature is also present in *O. salsburyi* [35] and species of the genus *Pseudogastromyzon* [31], indicating it may have conserved functional or structural significance within Cypriniformes or even a broader range of teleost fishes. Crucially, we detected a substantial number of non-canonical base pairs (G-U wobble pairs), ranging from 22 to 31 pairs, in the secondary structures of all tRNAs across all species. Although G-U pairs are less stable than G-C or A-T pairs, they still form hydrogen bonds of moderate strength in RNA structures. Such non-canonical pairing is also highly prevalent in the genus *Osteochilus* (e.g., 30 G-U pairs in *O. salsburyi* [30]) and the genus *Pseudogastromyzon* (e.g., 36 G-U pairs in *P. changtingensis tungpeiensis* [31]). Furthermore, given the absence of recombination in mitochondrial genomes, Lynch (1997) proposed that a certain level of base mispairing might help maintain functional plasticity of tRNAs within a limited mutational context, potentially even mitigating the effects of deleterious mutations by increasing mutational robustness [37].

Codon usage bias, a key feature of genomic evolution, reflects the balance between mutational pressure, natural selection, and genetic drift [38]. Analysis using the Relative Synonymous Codon Usage (RSCU) index revealed significant codon usage bias across all 13 PCGs, with a preference for codons ending in A or U at the third codon position. This pattern is consistent with the codon usage mode observed in the vast majority of teleost mitochondrial genomes; for example, RSCU values for NNU and NNA codons are also mostly greater than 1 in *O. salsburyi* [30]. This bias is considered primarily driven by the overall AT-biased mutational pressure of the genome. To further elucidate the influence of natural selection at the translational level on codon usage, this study identified 12 optimal codons (CUA, GUA, UCC, CCC, ACU, ACA, GCC, CAA, GAA, UGC, CGC, GGC) using *P. albomarginatus* as an example. These codons, enriched in highly expressed genes, are thought to match the abundance of corresponding tRNAs within the cell, thereby optimizing translation efficiency and enhancing protein synthesis rates [24]. Cross-species comparisons revealed that optimal codon sets exhibit both overlaps and differences among different taxa. For instance, *O. salsburyi* was reported to have 6 optimal codons (ACC, UAC, AAC, UGU, AGC, GGC) [30], while the most common optimal codons in the genus *Pseudogastromyzon* are CUA, GUA, CCA, CAA, GAA, AGC, GGC [31]. This pattern of shared and specific elements indicates that the evolution of optimal codons is influenced by universal functional constraints (e.g., translational efficiency) on one hand, and may also be associated with lineage-specific evolutionary histories, ecological adaptations, and effective population sizes on the other. Based on the correlation analysis between Effective Number of Codons (ENC) and GC3s content, along with PR2-plot analysis, we preliminarily infer that natural selection (likely translational selection) is the predominant force shaping the codon usage patterns in the mitochondrial PCGs of *Pseudobagrus* species. Although mutational pressure exerts a background influence through base composition, its role appears secondary. This conclusion resonates with findings in *O. salsburyi* [30] and the genus *Pseudogastromyzon* [31], suggesting that natural selection optimizing codon usage might be a common phenomenon in teleost mitochondrial genomes.

The BI and ML phylogenetic tree, constructed from the concatenated sequences of the 13 PCGs, all yielded a topology with high nodal support, clearly delineating the 15 *Pseudobagrus* species into two major clades. This robust phylogenetic framework provides crucial molecular evidence for resolving long-standing taxonomic controversies within the genus. Clade I encompassed the majority of the species (14 species) in this study and further resolved into several well-supported subclades. For example, *P. albomarginatus* and *P. tenuis* formed a sister group, which subsequently clustered with *P. brevicorpus*, *P. ussuriensis*, and other species in a stepwise manner. These relationships, supported by high posterior probabilities, suggest a relatively recent shared evolutionary history among them. More importantly, our results provide strong molecular phylogenetic support for the views proposed by Ng & Freyhof and Hardman, Ku, et al. [6,7,8]: namely, that the traditional genera *Pseudobagrus*, *Tachysurus*, and *Leiocassis* in East Asia form a monophyletic group, but each exhibits paraphyly or polyphyly, thereby supporting a systematic revision and merging of these groups. While the monophyly of *Pseudobagrus* as examined here is supported, a broader phylogenetic framework for the Bagridae requires validation with more comprehensive taxon sampling. Of particular note, The genetic relationship between *P. albomarginatus* and *P. ussuriensis* was relatively close. This aligns with the conclusions of Cheng et al. [10]. our phylogenetic analysis provided independent molecular evidence to support the phylogenetic relationship between *P. ussuriensis* and different species within the same genus. Furthermore, Cheng et al., in an integrative study of the *P. albomarginatus* species group, revealed the presence of cryptic species within *P. zhangfei* [10]. Although *P. zhangfei* was not included in the present study, this finding highlights the potential limitations of morphology-based species identification within *Pseudobagrus* [10,11]. Future research should incorporate more extensive population genetic sampling and genomic data to probe for potential cryptic diversity.

## 5. Conclusions

This study clarifies key scientific issues regarding the taxonomy and evolutionary biology of *Pseudobagrus*, a group of ecologically and economically critical East Asian freshwater fishes, by conducting comprehensive comparative and phylogenetic analyses of the complete mitochondrial genomes of 15 representative species. The mitochondrial genomes of these *Pseudobagrus* species exhibit structural conservation, with lengths ranging from 16,526 to 16,647 bp, without large-scale gene rearrangements—reflecting strong functional constraints to maintain core roles in cellular energy metabolism. At the genetic feature level, the *COI* gene uniquely uses GTG as the start codon (whereas the other 12 PCGs employ the canonical ATG), the stop codons of PCGs include complete TAA, TAG, and incomplete T-- (the latter completed via post-transcriptional polyadenylation); additionally, 12, 9, and 15 optimal codons are identified in *P. albomarginatus*, *P. koreanus*, and *P. brevicorpus*, respectively, with a preference for NNU/NNA codons primarily driven by natural selection. Phylogenetic analysis based on concatenated PCGs clearly divides the 15 species into two major clades (14 species in Clade I and one species in Clade II). Collectively, these findings provide a robust molecular basis for species delimitation and systematic revision of *Pseudobagrus*, and offer essential genetic support for promoting the sustainable development of aquaculture, protecting freshwater ecosystems.

## Figures and Tables

**Table 1:** Composition and skewness in mitogenomes of the 15 *Pseudobagrus* species.

Species	Length/bp	Base Composition/%							
		A	C	G	T	A + T	C + G	AT-Skew	GC-Skew
*Pseudobagrus albomarginatus*	16,533	31.78	26.35	14.86	27.00	58.78	41.21	0.08132	−0.27882
*Pseudobagrus brachyrhabdion*	16,532	31.02	26.39	15.55	27.05	58.07	41.94	0.06837	−0.25846
*Pseudobagrus brevicaudatus*	16,533	31.65	26.59	14.95	26.82	58.47	41.54	0.08261	−0.28021
*Pseudobagrus brevicorpus*	16,526	30.82	27.99	15.45	25.74	56.56	43.44	0.08982	−0.28867
*Pseudobagrus emarginatus*	16,534	31.55	26.79	14.91	26.75	58.30	41.70	0.08233	−0.28489
*Pseudobagrus gracilis*	16,533	31.03	26.31	15.52	27.14	58.17	41.83	0.06687	−0.25795
*Pseudobagrus koreanus*	16,532	30.96	27.60	15.30	26.14	57.10	42.90	0.08441	−0.28671
*Pseudobagrus medianalis*	16,647	30.91	28.00	15.38	25.70	56.61	43.38	0.09203	−0.29092
*Pseudobagrus ondon*	16,534	31.06	27.97	15.24	25.72	56.78	43.21	0.09405	−0.29461
*Pseudobagrus pratti*	16,533	31.55	26.78	14.91	26.76	58.31	41.69	0.08215	−0.28472
*Pseudobagrus tenuis*	16,535	31.76	26.38	14.88	26.99	58.75	41.26	0.08119	−0.27872
*Pseudobagrus tokiensis*	16,529	30.91	27.85	15.33	25.91	56.82	43.18	0.088	−0.28995
*Pseudobagrus trilineatus*	16,535	31.56	27.13	14.83	26.48	58.04	41.96	0.08753	−0.29314
*Pseudobagrus truncatu*	16,533	31.56	26.78	14.93	26.73	58.29	41.71	0.08286	−0.2841
*Pseudobagrus ussuriensis*	16,536	31.79	26.50	14.87	26.84	58.63	41.37	0.08443	−0.28112
Mean value	16,540.33	31.33	27.03	15.13	26.52	57.85	42.15	0.08314	−0.28229

**Table 2:** Analysis of codon adaption index, effective number of codons, and GC content of the 15 *Pseudobagrus* mitogenomes.

Species	CAI	ENC	GC3s	GC	GC1	GC2	GC3
*Pseudobagrus albomarginatus*	0.169	55.950	0.429	0.430	50.51%	40.58%	31.68%
*Pseudobagrus brachyrhabdion*	0.161	55.740	0.426	0.437	51.03%	40.73%	34.20%
*Pseudobagrus brevicaudatus*	0.165	55.020	0.408	0.430	50.54%	40.53%	32.52%
*Pseudobagrus brevicorpus*	0.158	55.840	0.429	0.449	51.00%	40.76%	39.34%
*Pseudobagrus emarginatus*	0.159	56.940	0.437	0.451	50.59%	40.45%	33.83%
*Pseudobagrus gracilis*	0.154	55.320	0.400	0.435	50.91%	40.58%	33.94%
*Pseudobagrus koreanus*	0.162	55.720	0.430	0.447	50.81%	40.81%	37.76%
*Pseudobagrus medianalis*	0.150	55.070	0.420	0.448	51.23%	40.73%	38.92%
*Pseudobagrus ondon*	0.166	54.990	0.421	0.434	51.19%	40.63%	38.61%
*Pseudobagrus pratti*	0.167	55.280	0.421	0.434	50.75%	40.45%	33.70%
*Pseudobagrus tenuis*	0.170	55.950	0.432	0.430	50.49%	40.56%	31.86%
*Pseudobagrus tokiensis*	0.164	55.660	0.439	0.450	51.33%	40.89%	38.58%
*Pseudobagrus trilineatus*	0.161	53.960	0.412	0.434	49.79%	40.41%	35.61%
*Pseudobagrus truncatus*	0.168	55.300	0.423	0.434	50.72%	40.48%	33.86%
*Pseudobagrus ussuriensis*	0.147	53.460	0.368	0.425	50.67%	40.43%	32.13%
Mean values	0.161	55.347	0.420	0.438	50.77%	40.60%	35.10%

## Data Availability

The raw data supporting the conclusions of this article will be made available by the authors on request.

## Supplementary Materials

The following supporting information can be downloaded at: https://www.mdpi.com/article/10.3390/ani16020279/s1, Figure S1: Summary of complete mitochondrial gene/element features of the 15 *Pseudobagrus* species. Figure S2: Schematic diagram of tRNAs secondary structures in the *Pseudobagrus* mitogenomes. Table S1: Mitogenome characteristic of the 15 *Pseudobagrus* species. Table S2: The codon number and relative synonymous codon usage (RSCU) in mitochondrial protein coding genes of the 15 *Pseudobagrus* species. Table S3: Optimal codons analysis of the 13PCGs of the 15 *Pseudobagrus* species. Table S4: Analysis of codon adaption index (CAI) and GC content of the 13PCGs of the 15 *Pseudobagrus* species.
