# Supplementary material for: Identified Optimal Codons and Phylogenetic Relationship in Pseudobagrus Species Based on Complete Mitogenomes
Source: Animals (Basel). 2026 Jan 16;16(2):279. doi: 10.3390/ani16020279 (PMC12837665; doi:10.3390/ani16020279)
Supplement: Supplementary file 1 [file animals-16-00279-s001.zip › Figure S1.pdf]

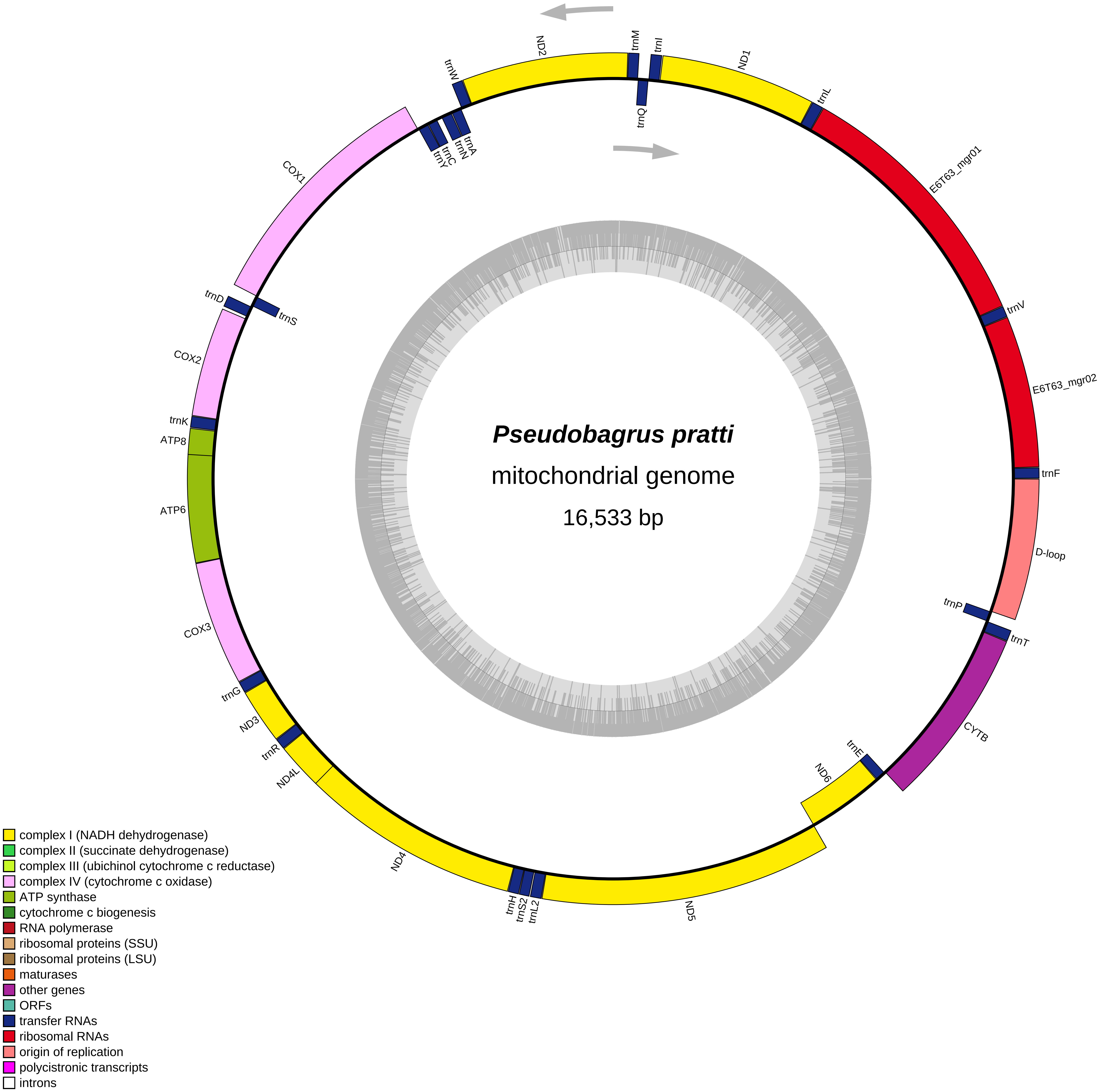

Pseudobagrus pratti
mitochondrial genome
16,533 bp
ND2
trnM
trnI
ND1
trnW
trnQ
trnL
trnA
trnN
trnC
trnY
E6T63_mgr01
COX1
trnD
trnS
trnV
COX2
E6T63_mgr02
trnK
ATP8
trnF
ATP6
D-loop
trnP
COX3
trnT
trnG
ND3
CYTB
trnE
trnR
ND6
ND4L
ND4
trnH
trnS2
trnL2
ND5
complex I (NADH dehydrogenase)
complex II (succinate dehydrogenase)
complex III (ubichinol cytochrome c reductase)
complex IV (cytochrome c oxidase)
ATP synthase
cytochrome c biogenesis
RNA polymerase
ribosomal proteins (SSU)
ribosomal proteins (LSU)
maturases
other genes
ORFs
transfer RNAs
ribosomal RNAs
origin of replication
polycistronic transcripts
introns

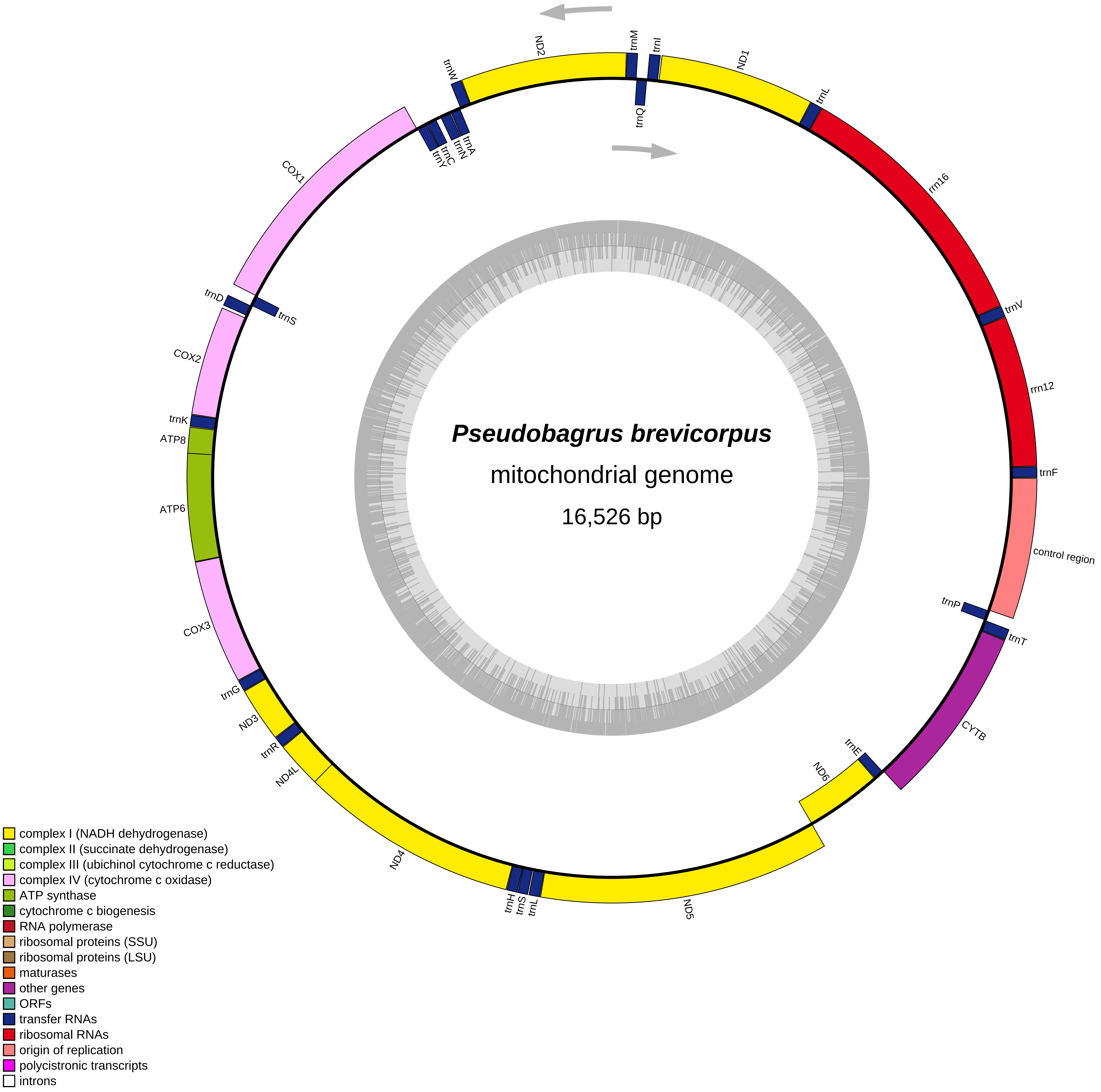

Pseudobagrus brevicorpus
mitochondrial genome
16,526 bp
ND2
trnM
trnI
ND1
trnL
trnW
trnQ
trnA
trnN
trnC
trnY
rrn16
COX1
trnV
trnD
trnS
rrn12
COX2
trnK
ATP8
trnF
ATP6
control region
trnP
COX3
trnT
trnG
ND3
CYTB
trnE
trnR
ND6
ND4L
ND4
trnH
trnS
trnL
ND5
complex I (NADH dehydrogenase)
complex II (succinate dehydrogenase)
complex III (ubichinol cytochrome c reductase)
complex IV (cytochrome c oxidase)
ATP synthase
cytochrome c biogenesis
RNA polymerase
ribosomal proteins (SSU)
ribosomal proteins (LSU)
maturases
other genes
ORFs
transfer RNAs
ribosomal RNAs
origin of replication
polycistronic transcripts
introns

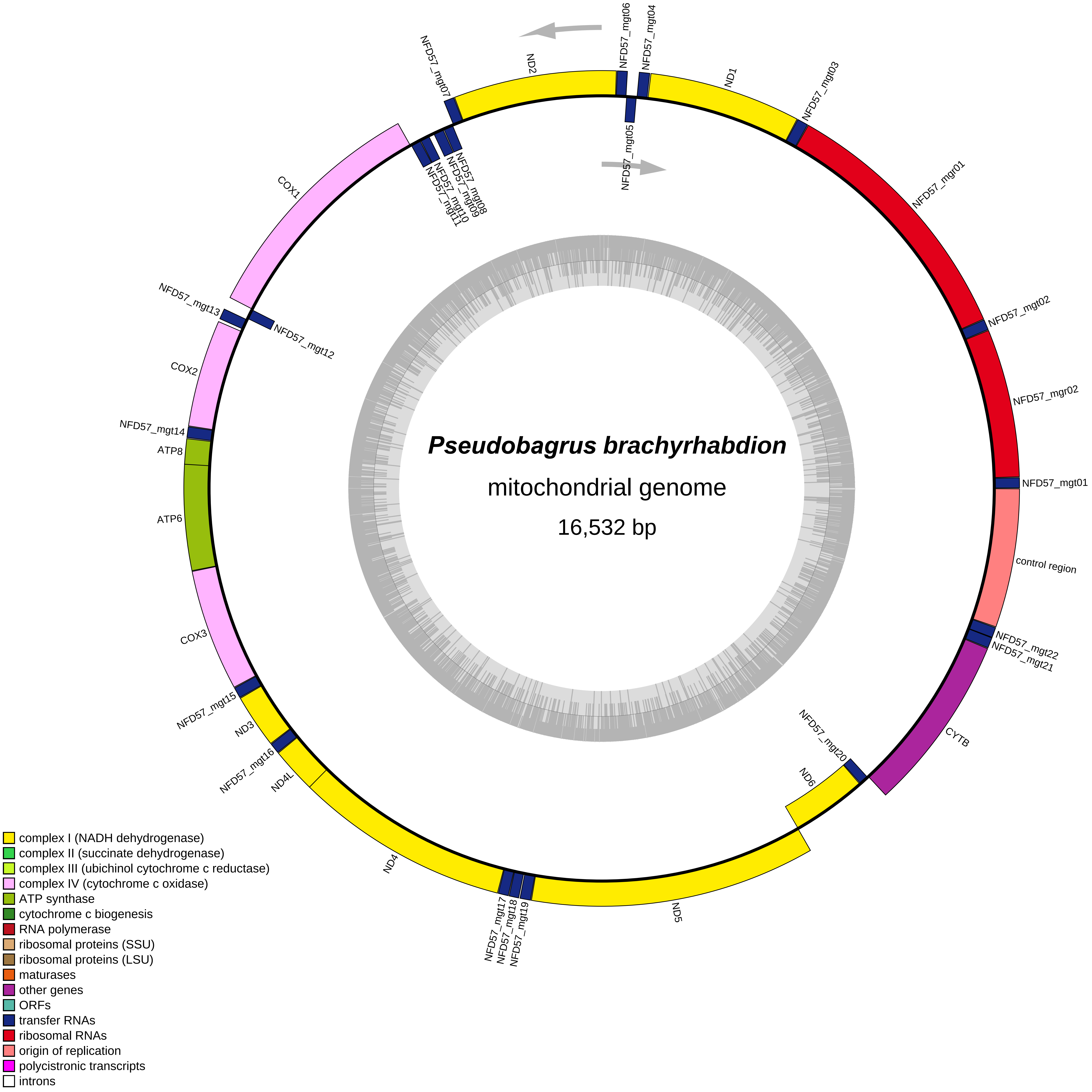

Pseudobagrus brachyrhabdion
mitochondrial genome
16,532 bp
NFD57_mgt06
NFD57_mgt04
ND1
NFD57_mgt03
NFD57_mgr01
NFD57_mgt02
NFD57_mgr02
NFD57_mgt01
control region
NFD57_mgt22
NFD57_mgt21
CYTB
NFD57_mgt20
ND6
ND5
NFD57_mgt17
NFD57_mgt18
NFD57_mgt19
ND4
ND4L
NFD57_mgt16
ND3
NFD57_mgt15
COX3
ATP6
ATP8
NFD57_mgt14
COX2
NFD57_mgt13
NFD57_mgt12
COX1
NFD57_mgt11
NFD57_mgt10
NFD57_mgt09
NFD57_mgt08
NFD57_mgt07
ND2
NFD57_mgt05
complex I (NADH dehydrogenase)
complex II (succinate dehydrogenase)
complex III (ubichinol cytochrome c reductase)
complex IV (cytochrome c oxidase)
ATP synthase
cytochrome c biogenesis
RNA polymerase
ribosomal proteins (SSU)
ribosomal proteins (LSU)
maturases
other genes
ORFs
transfer RNAs
ribosomal RNAs
origin of replication
polycistronic transcripts
introns

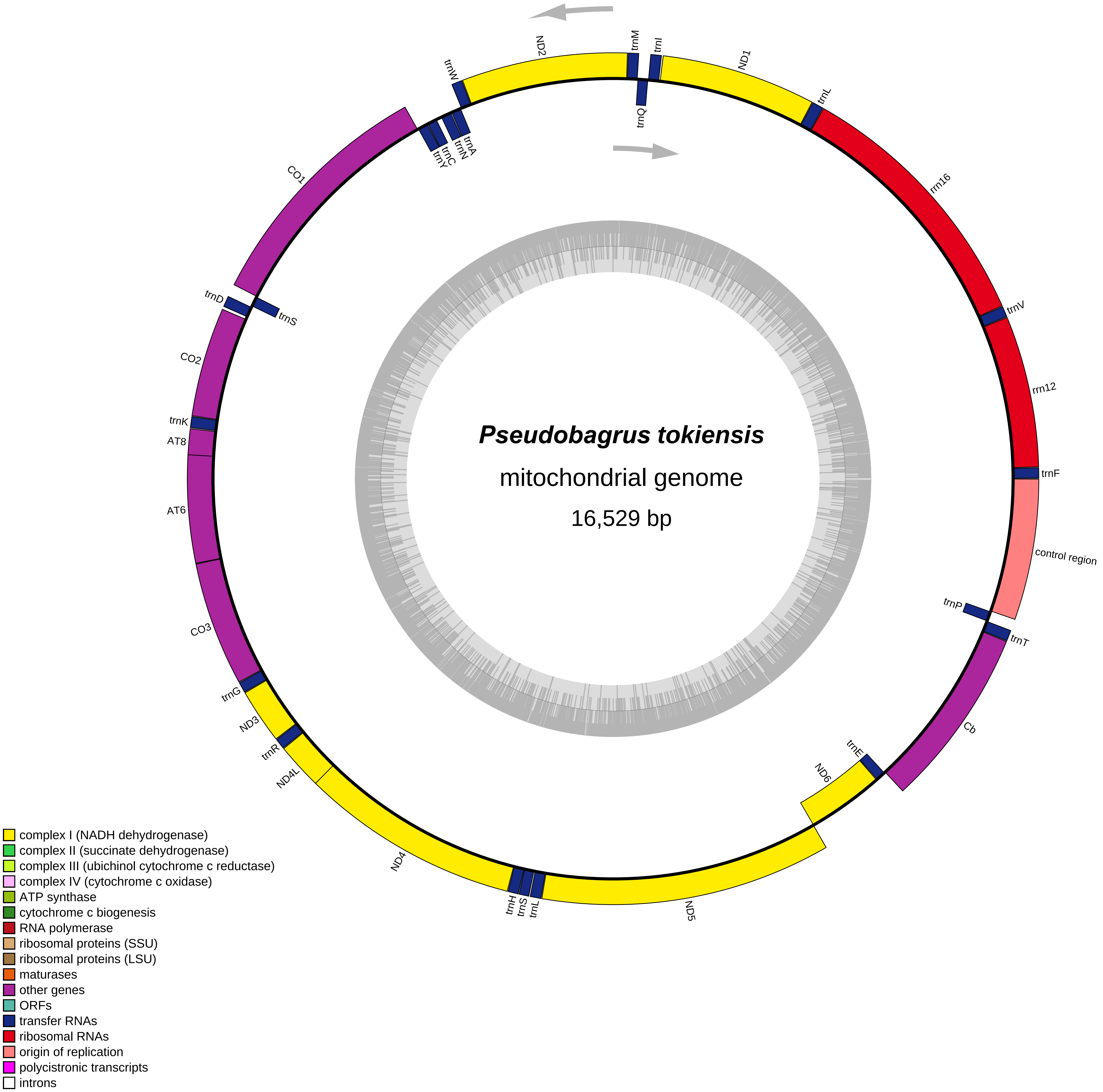
Pseudobagrus tokiensis
mitochondrial genome
16,529 bp
trnM
trnI
ND1
trnL
rrn16
trnV
rrn12
trnF
control region
trnP
trnT
Cb
trnE
ND6
ND5
trnL
trnS
trnH
ND4
ND4L
trnR
ND3
trnG
CO3
AT6
AT8
trnK
CO2
trnD
trnS
CO1
trnY
trnC
trnN
trnA
trnW
ND2
trnQ
complex I (NADH dehydrogenase)
complex II (succinate dehydrogenase)
complex III (ubichinol cytochrome c reductase)
complex IV (cytochrome c oxidase)
ATP synthase
cytochrome c biogenesis
RNA polymerase
ribosomal proteins (SSU)
ribosomal proteins (LSU)
maturases
other genes
ORFs
transfer RNAs
ribosomal RNAs
origin of replication
polycistronic transcripts
introns

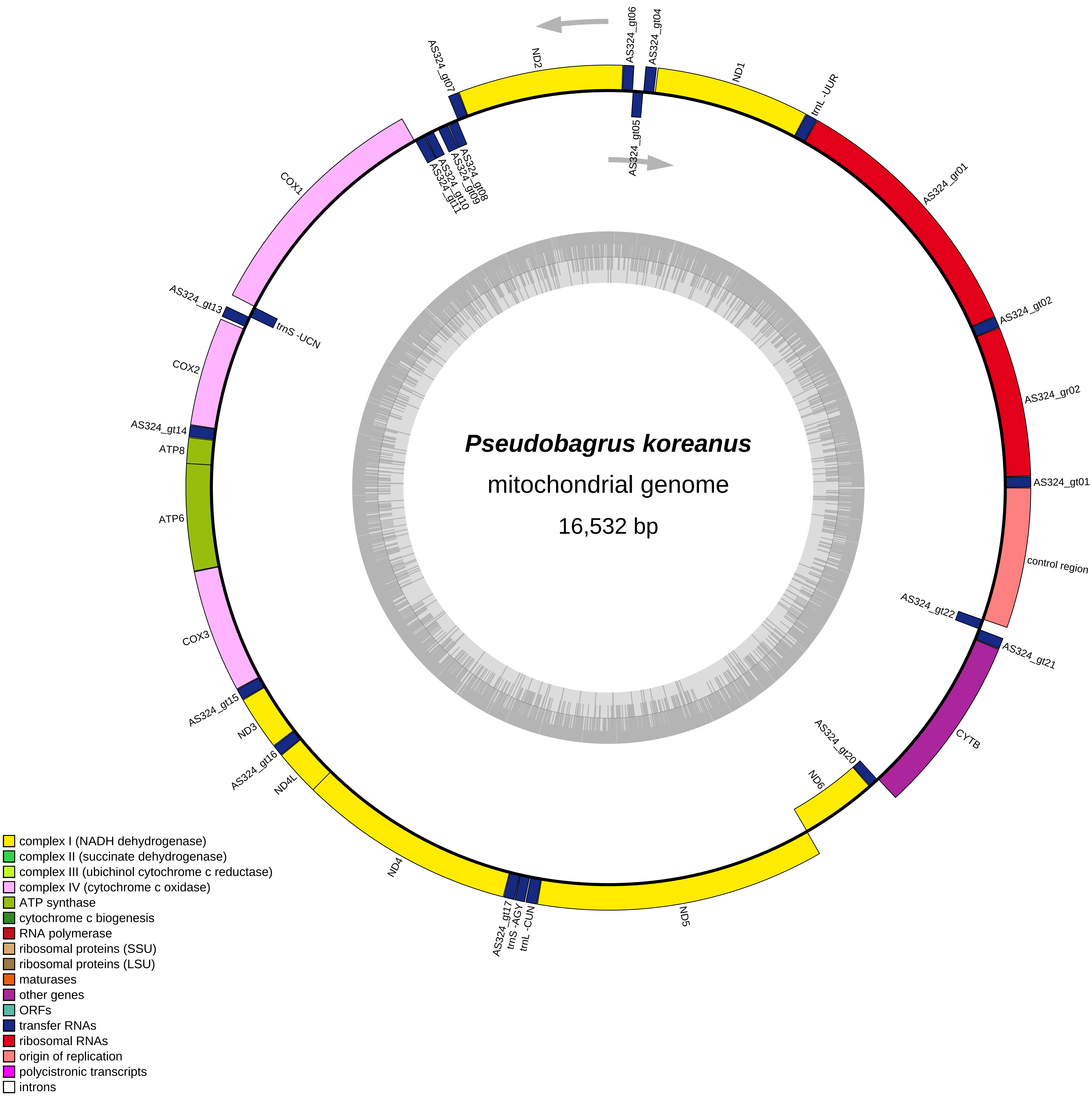

Pseudobagrus koreanus
mitochondrial genome
16,532 bp
ND2
AS324_gt06
AS324_gt04
ND1
trnL -UUR
AS324_gt07
AS324_gt05
AS324_gt08
AS324_gt09
AS324_gt10
AS324_gt11
COX1
AS324_gr01
AS324_gt02
AS324_gr02
AS324_gt01
AS324_gt13
trnS -UCN
COX2
AS324_gt14
ATP8
ATP6
control region
AS324_gt22
AS324_gt21
COX3
CYTB
AS324_gt15
ND3
AS324_gt20
AS324_gt16
ND4L
ND6
ND4
AS324_gt17
trnS -AGY
trnL -CUN
ND5
complex I (NADH dehydrogenase)
complex II (succinate dehydrogenase)
complex III (ubichinol cytochrome c reductase)
complex IV (cytochrome c oxidase)
ATP synthase
cytochrome c biogenesis
RNA polymerase
ribosomal proteins (SSU)
ribosomal proteins (LSU)
maturases
other genes
ORFs
transfer RNAs
ribosomal RNAs
origin of replication
polycistronic transcripts
introns

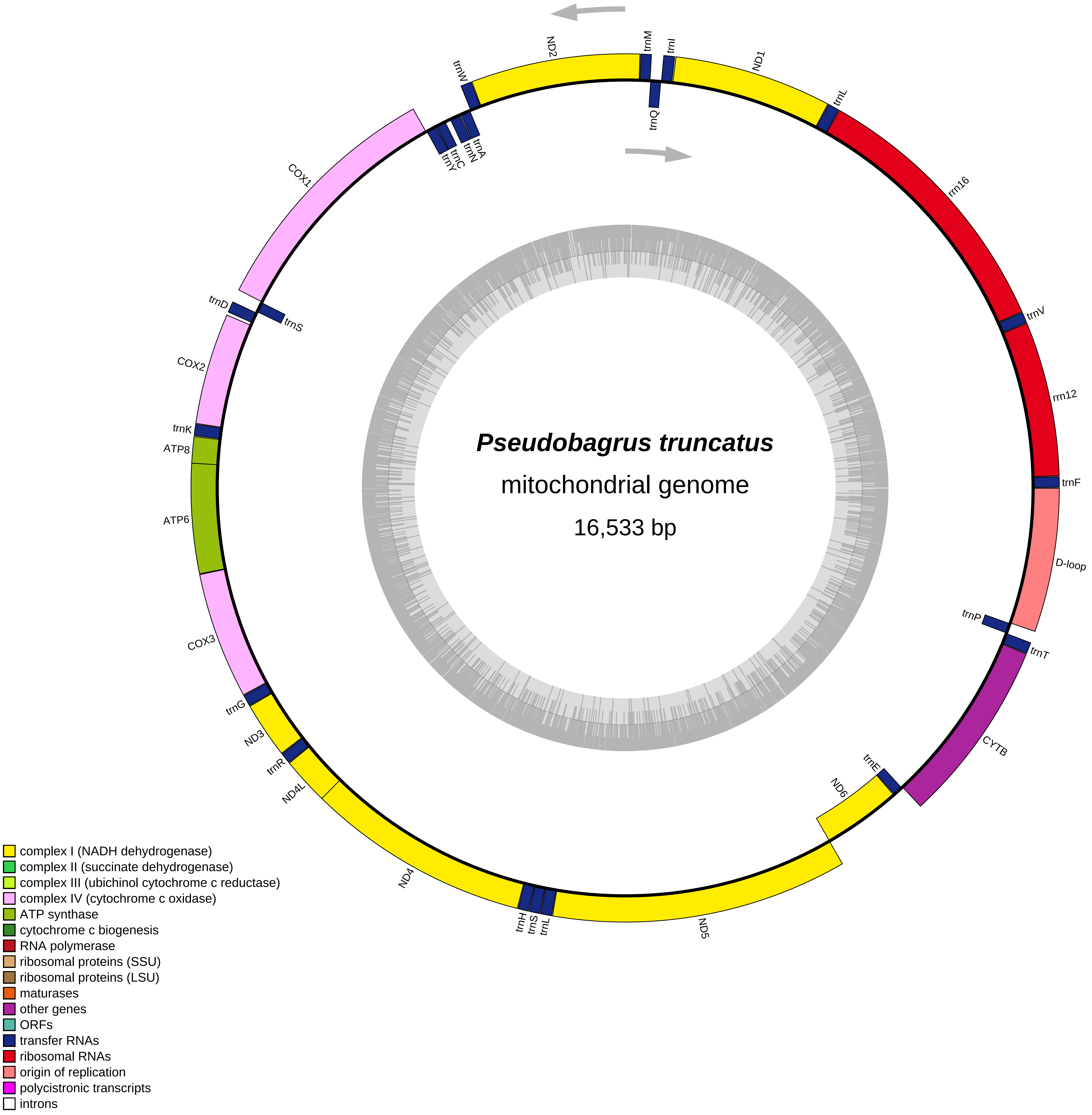

Pseudobagrus truncatus
mitochondrial genome
16,533 bp
trnM
trnI
ND1
trnL
rrn16
trnV
rrn12
trnF
D-loop
trnP
trnT
CYTB
trnE
ND6
ND5
trnL
trnS
trnH
ND4
ND4L
trnR
ND3
trnG
COX3
ATP6
ATP8
trnK
COX2
trnD
trnS
COX1
trnY
trnC
trnN
trnA
trnW
ND2
trnQ
complex I (NADH dehydrogenase)
complex II (succinate dehydrogenase)
complex III (ubichinol cytochrome c reductase)
complex IV (cytochrome c oxidase)
ATP synthase
cytochrome c biogenesis
RNA polymerase
ribosomal proteins (SSU)
ribosomal proteins (LSU)
maturases
other genes
ORFs
transfer RNAs
ribosomal RNAs
origin of replication
polycistronic transcripts
introns

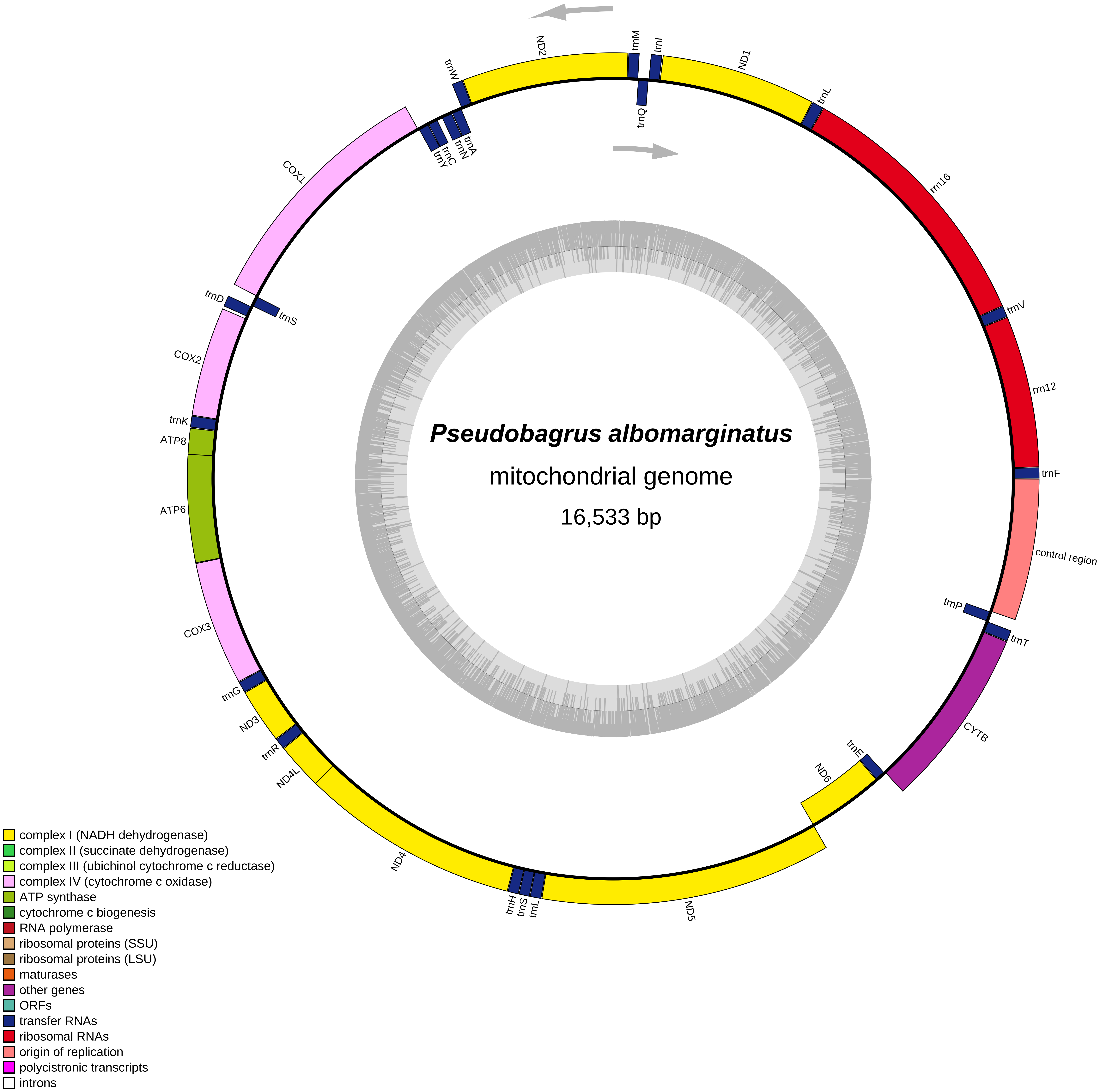

Pseudobagrus albomarginatus
mitochondrial genome
16,533 bp
ND2
trnM
trnI
ND1
trnW
trnQ
trnL
trnA
trnN
trnC
trnY
COX1
rrn16
trnD
trnS
trnV
COX2
rrn12
trnK
ATP8
trnF
ATP6
control region
trnP
COX3
trnT
trnG
ND3
CYTB
trnR
trnE
ND4L
ND6
ND4
trnH
trnS
trnL
ND5
complex I (NADH dehydrogenase)
complex II (succinate dehydrogenase)
complex III (ubichinol cytochrome c reductase)
complex IV (cytochrome c oxidase)
ATP synthase
cytochrome c biogenesis
RNA polymerase
ribosomal proteins (SSU)
ribosomal proteins (LSU)
maturases
other genes
ORFs
transfer RNAs
ribosomal RNAs
origin of replication
polycistronic transcripts
introns

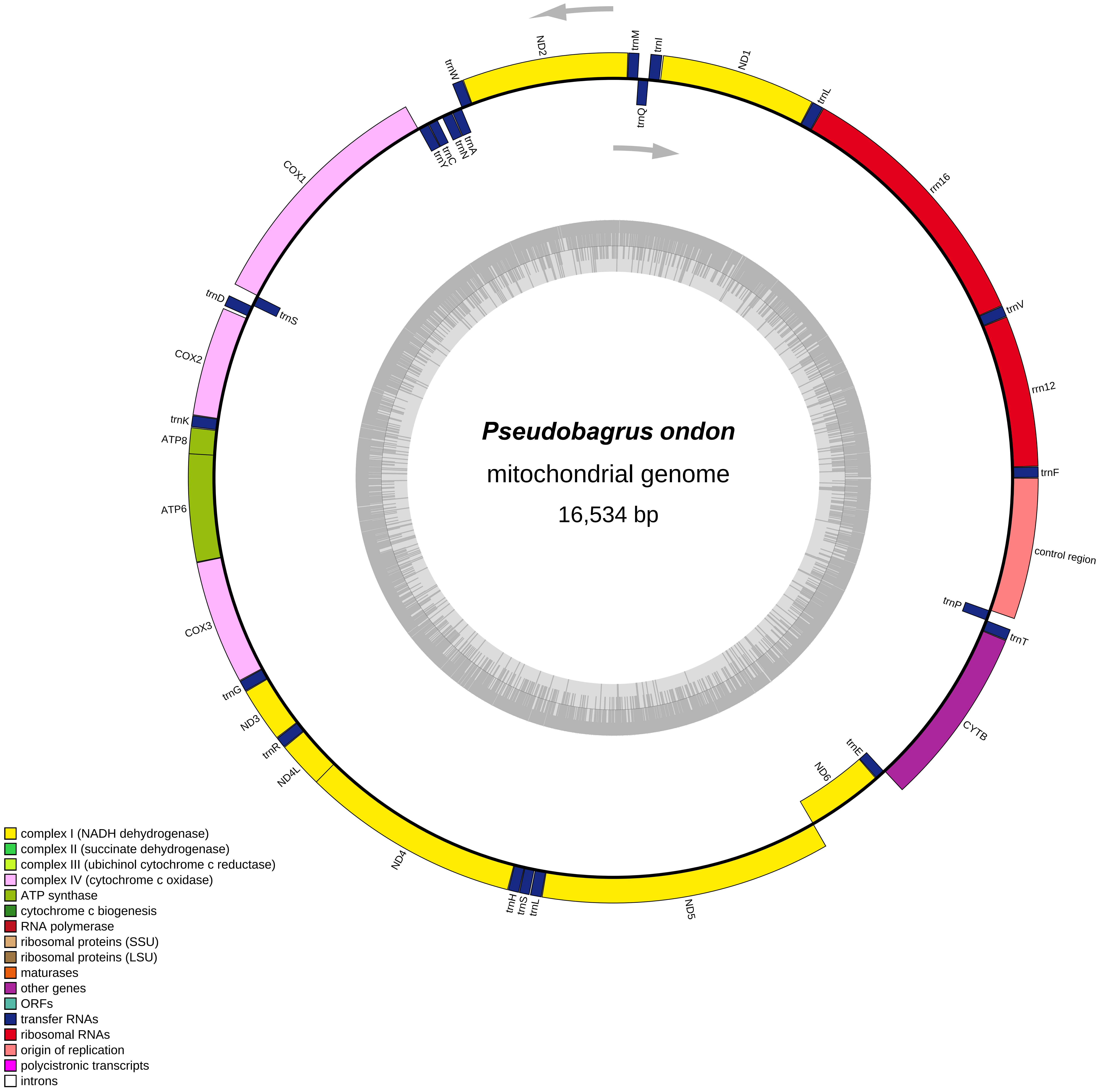

Pseudobagrus ondon
mitochondrial genome
16,534 bp
trnM
trnI
ND1
trnL
rrn16
trnV
rrn12
trnF
control region
trnP
trnT
CYTB
trnE
ND6
ND5
trnL
trnS
trnH
ND4
ND4L
trnR
ND3
trnG
COX3
ATP6
ATP8
trnK
COX2
trnD
trnS
COX1
trnY
trnC
trnN
trnA
trnW
ND2
trnQ
complex I (NADH dehydrogenase)
complex II (succinate dehydrogenase)
complex III (ubichinol cytochrome c reductase)
complex IV (cytochrome c oxidase)
ATP synthase
cytochrome c biogenesis
RNA polymerase
ribosomal proteins (SSU)
ribosomal proteins (LSU)
maturases
other genes
ORFs
transfer RNAs
ribosomal RNAs
origin of replication
polycistronic transcripts
introns

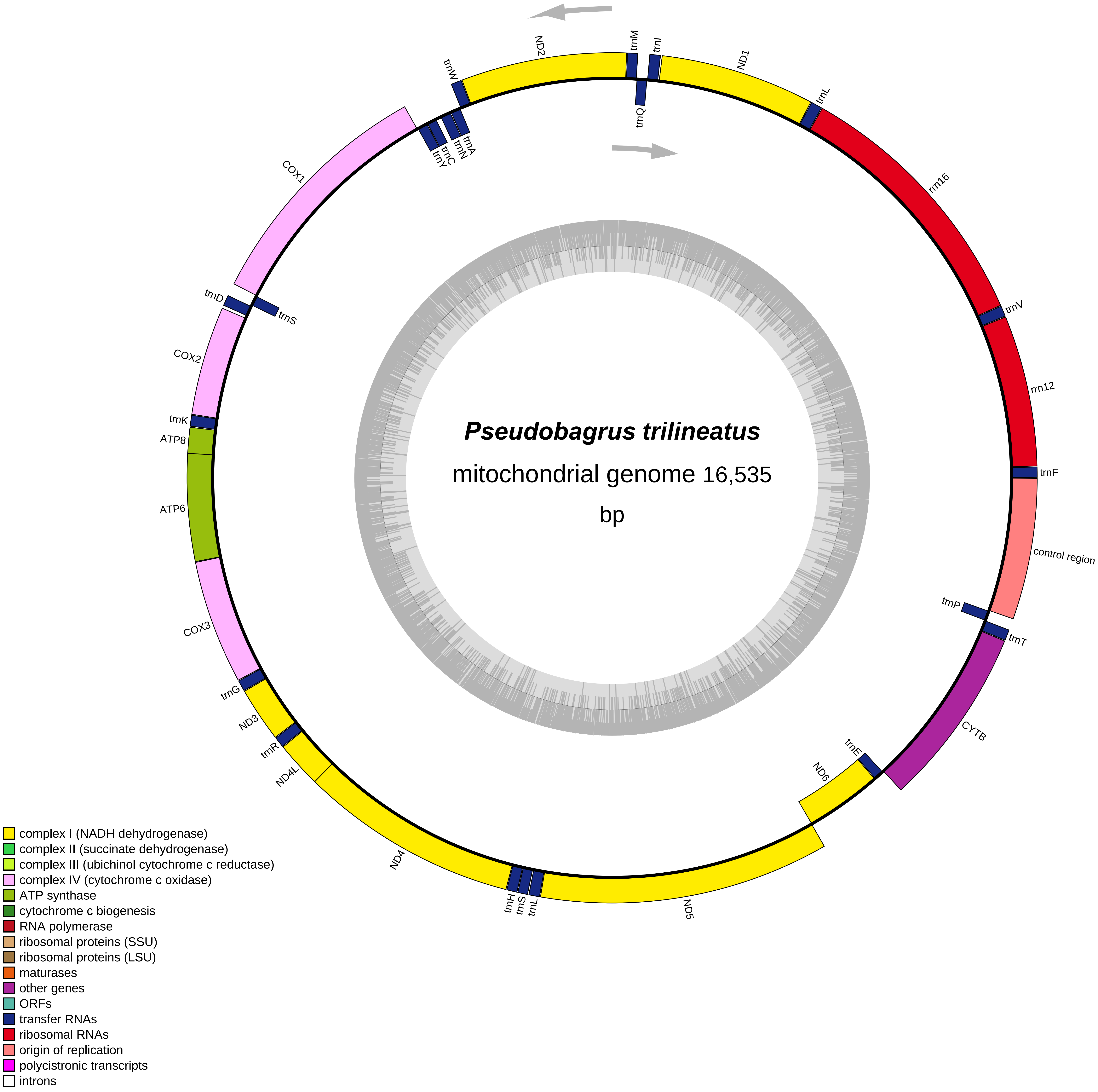

Pseudobagrus trilineatus
mitochondrial genome 16,535 bp
trnM
trnI
ND2
ND1
trnW
trnQ
trnL
trnA
trnN
trnC
trnY
COX1
rrn16
trnD
trnS
trnV
COX2
rrn12
trnK
ATP8
trnF
ATP6
control region
trnP
trnT
COX3
trnG
CYTB
ND3
trnE
trnR
ND6
ND4L
ND4
trnH
trnS
trnL
ND5
complex I (NADH dehydrogenase)
complex II (succinate dehydrogenase)
complex III (ubichinol cytochrome c reductase)
complex IV (cytochrome c oxidase)
ATP synthase
cytochrome c biogenesis
RNA polymerase
ribosomal proteins (SSU)
ribosomal proteins (LSU)
maturases
other genes
ORFs
transfer RNAs
ribosomal RNAs
origin of replication
polycistronic transcripts
introns

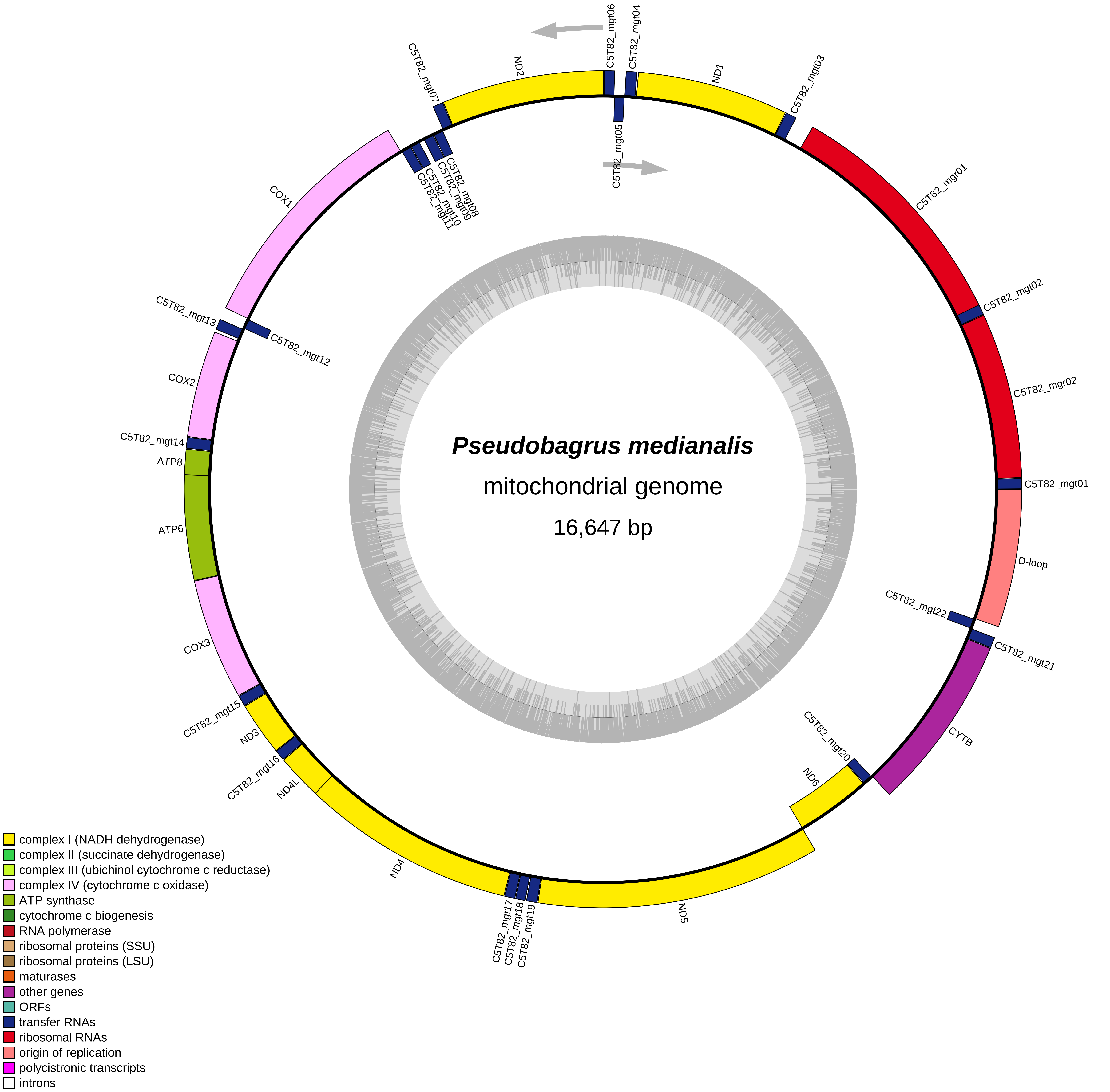

Pseudobagrus medianalis
mitochondrial genome
16,647 bp
ND2
C5T82_mgt06
C5T82_mgt04
ND1
C5T82_mgt03
C5T82_mgt07
C5T82_mgt05
C5T82_mgt08
C5T82_mgt09
C5T82_mgt10
C5T82_mgt11
C5T82_mgr01
COX1
C5T82_mgt02
C5T82_mgt13
C5T82_mgt12
C5T82_mgr02
COX2
C5T82_mgt14
ATP8
C5T82_mgt01
ATP6
D-loop
C5T82_mgt22
COX3
C5T82_mgt21
C5T82_mgt15
C5T82_mgt20
ND3
CYTB
C5T82_mgt16
ND6
ND4L
ND4
ND5
C5T82_mgt17
C5T82_mgt18
C5T82_mgt19
complex I (NADH dehydrogenase)
complex II (succinate dehydrogenase)
complex III (ubichinol cytochrome c reductase)
complex IV (cytochrome c oxidase)
ATP synthase
cytochrome c biogenesis
RNA polymerase
ribosomal proteins (SSU)
ribosomal proteins (LSU)
maturases
other genes
ORFs
transfer RNAs
ribosomal RNAs
origin of replication
polycistronic transcripts
introns

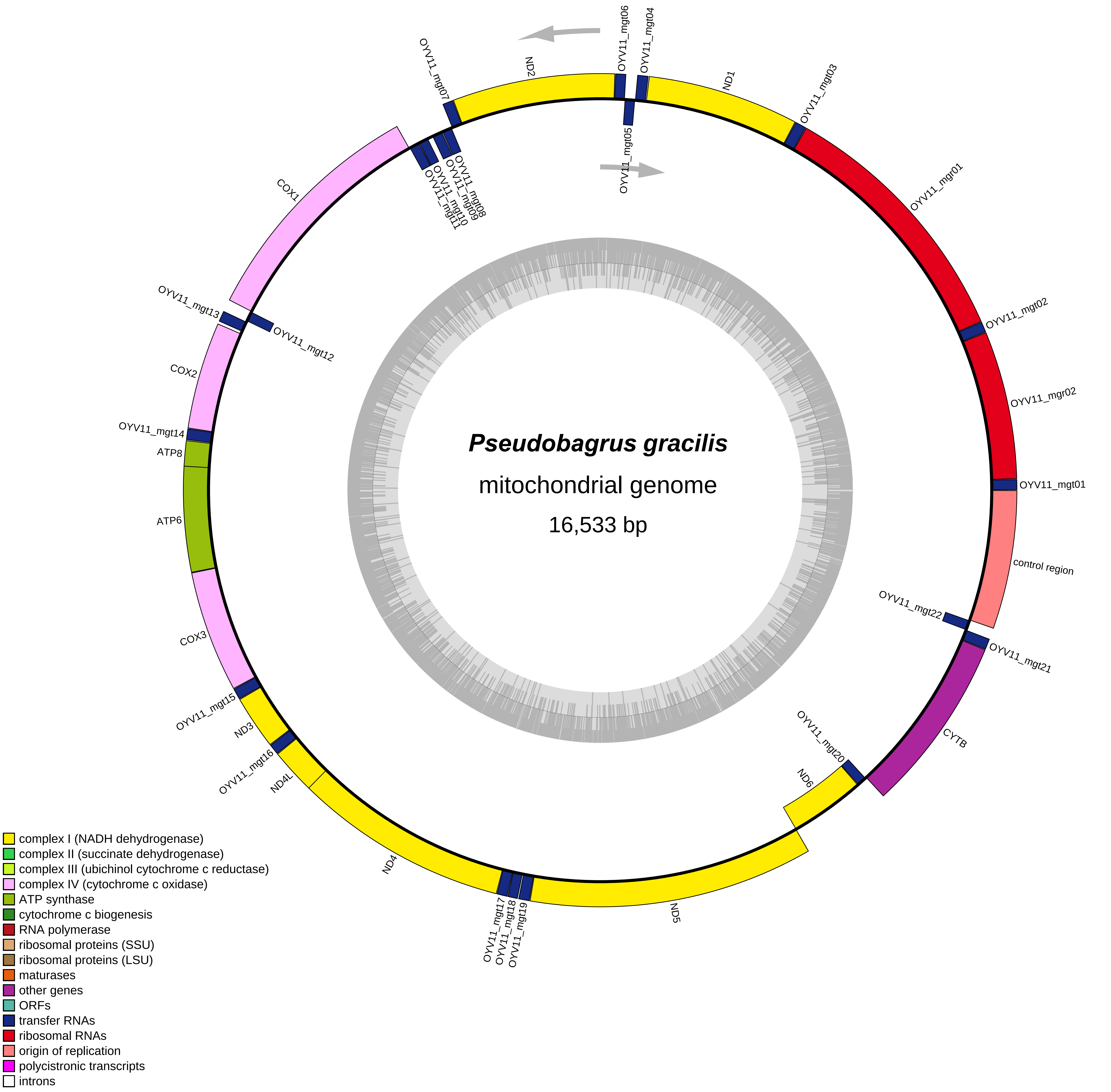

Pseudobagrus gracilis
mitochondrial genome
16,533 bp
ND1
ND2
COX1
COX2
ATP8
ATP6
COX3
ND3
ND4L
ND4
ND5
ND6
CYTB
control region
OYV11_mgr01
OYV11_mgr02
OYV11_mgt01
OYV11_mgt02
OYV11_mgt03
OYV11_mgt04
OYV11_mgt05
OYV11_mgt06
OYV11_mgt07
OYV11_mgt08
OYV11_mgt09
OYV11_mgt10
OYV11_mgt11
OYV11_mgt12
OYV11_mgt13
OYV11_mgt14
OYV11_mgt15
OYV11_mgt16
OYV11_mgt17
OYV11_mgt18
OYV11_mgt19
OYV11_mgt20
OYV11_mgt21
OYV11_mgt22
complex I (NADH dehydrogenase)
complex II (succinate dehydrogenase)
complex III (ubichinol cytochrome c reductase)
complex IV (cytochrome c oxidase)
ATP synthase
cytochrome c biogenesis
RNA polymerase
ribosomal proteins (SSU)
ribosomal proteins (LSU)
maturases
other genes
ORFs
transfer RNAs
ribosomal RNAs
origin of replication
polycistronic transcripts
introns

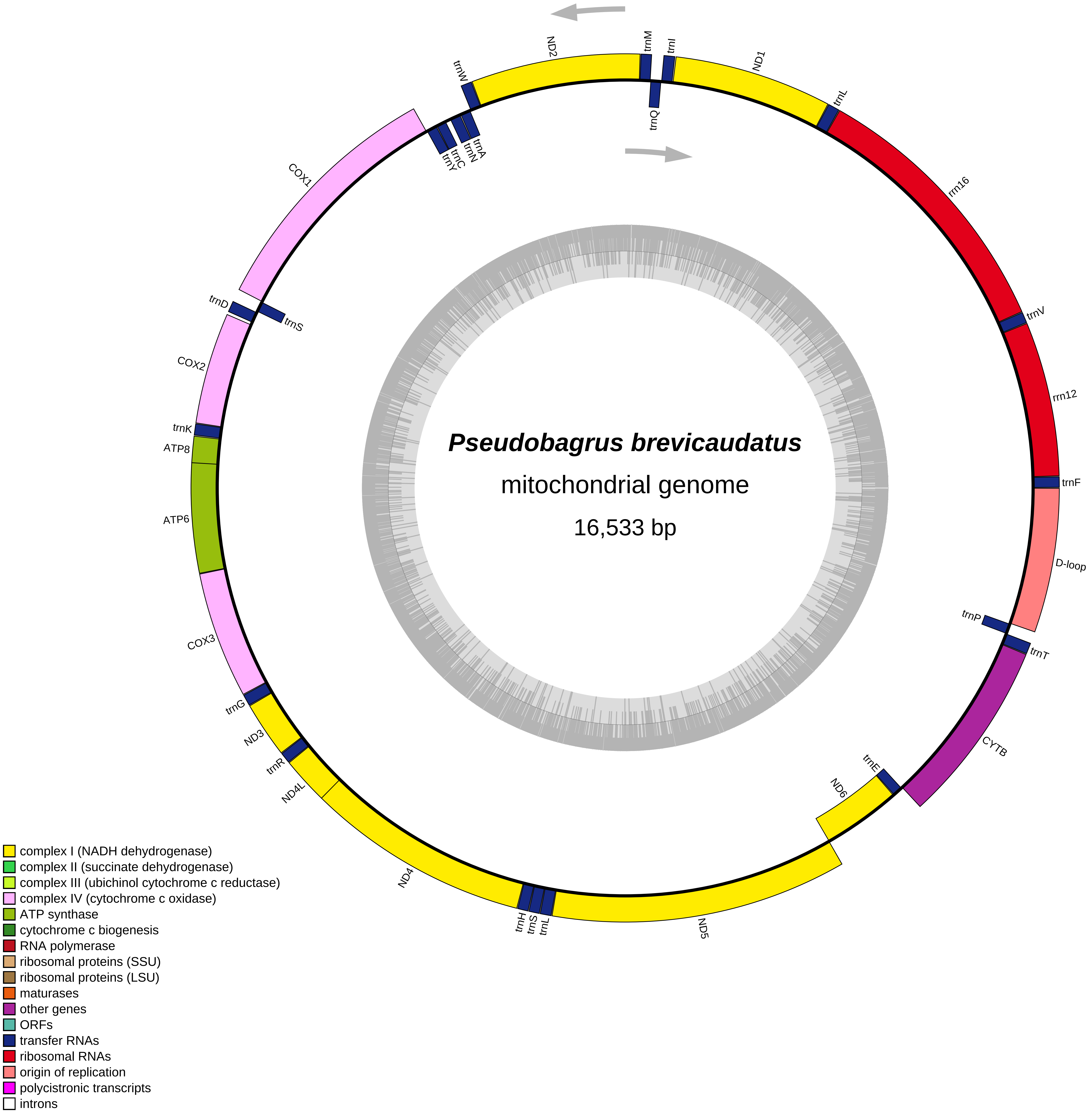

Pseudobagrus brevicaudatus
mitochondrial genome
16,533 bp
ND2
trnM
trnI
ND1
trnW
trnQ
trnL
trnA
trnN
trnC
trnY
rrn16
COX1
trnV
trnD
trnS
rrn12
COX2
trnK
ATP8
trnF
ATP6
D-loop
trnP
trnT
COX3
trnG
ND3
CYTB
trnE
trnR
ND6
ND4L
ND4
trnH
trnS
trnL
ND5
complex I (NADH dehydrogenase)
complex II (succinate dehydrogenase)
complex III (ubichinol cytochrome c reductase)
complex IV (cytochrome c oxidase)
ATP synthase
cytochrome c biogenesis
RNA polymerase
ribosomal proteins (SSU)
ribosomal proteins (LSU)
maturases
other genes
ORFs
transfer RNAs
ribosomal RNAs
origin of replication
polycistronic transcripts
introns

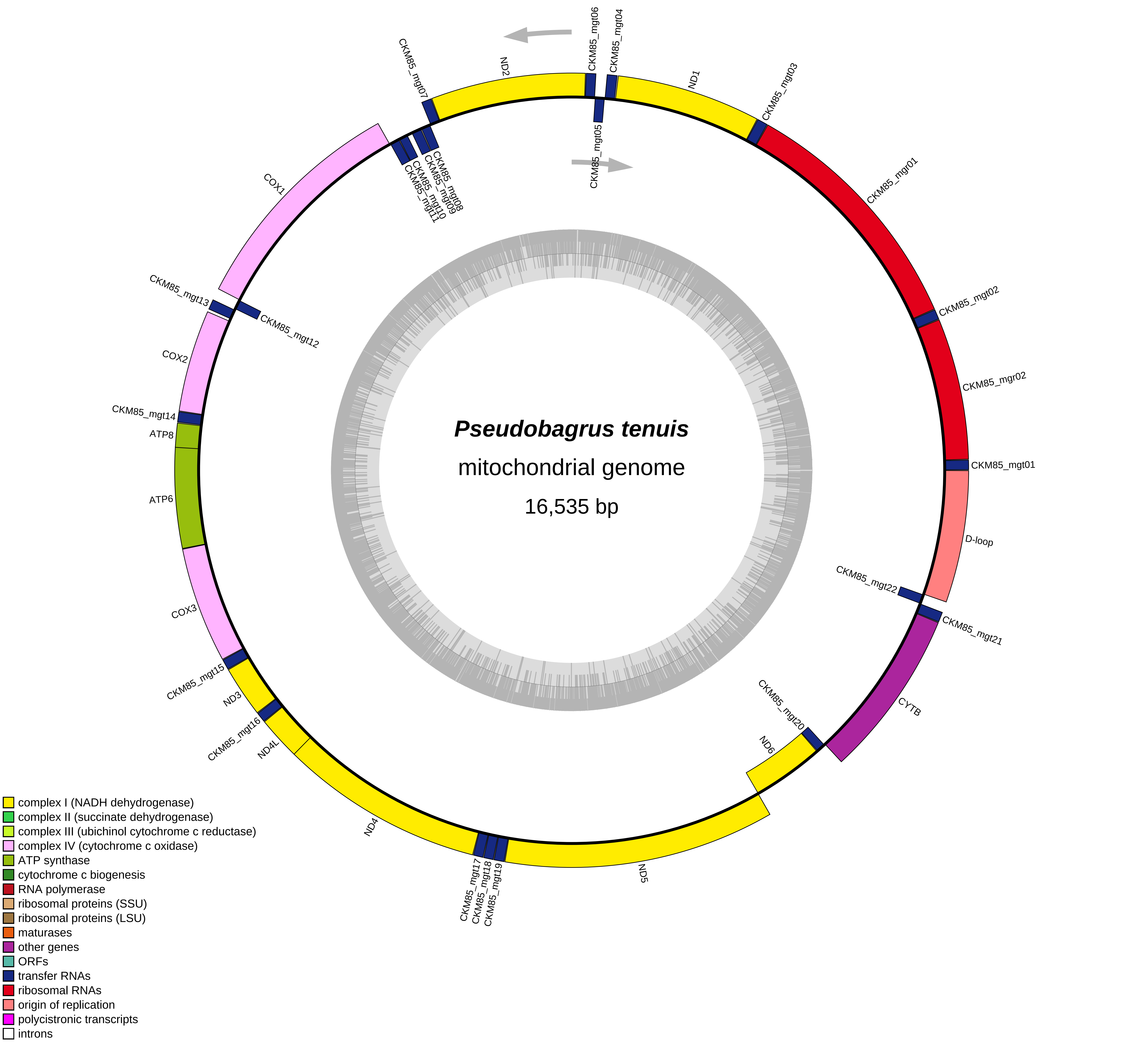
Pseudobagrus tenuis
mitochondrial genome
16,535 bp
ND1
ND2
CKM85_mgt01
CKM85_mgt02
CKM85_mgt03
CKM85_mgt04
CKM85_mgt05
CKM85_mgt06
CKM85_mgt07
CKM85_mgt08
CKM85_mgt09
CKM85_mgt10
CKM85_mgt11
CKM85_mgt12
CKM85_mgt13
CKM85_mgt14
CKM85_mgt15
CKM85_mgt16
CKM85_mgt17
CKM85_mgt18
CKM85_mgt19
CKM85_mgt20
CKM85_mgt21
CKM85_mgt22
CKM85_mgr01
CKM85_mgr02
D-loop
CYTB
ND6
ND5
ND4
ND4L
ND3
COX3
ATP6
ATP8
COX2
COX1
complex I (NADH dehydrogenase)
complex II (succinate dehydrogenase)
complex III (ubichinol cytochrome c reductase)
complex IV (cytochrome c oxidase)
ATP synthase
cytochrome c biogenesis
RNA polymerase
ribosomal proteins (SSU)
ribosomal proteins (LSU)
maturases
other genes
ORFs
transfer RNAs
ribosomal RNAs
origin of replication
polycistronic transcripts
introns

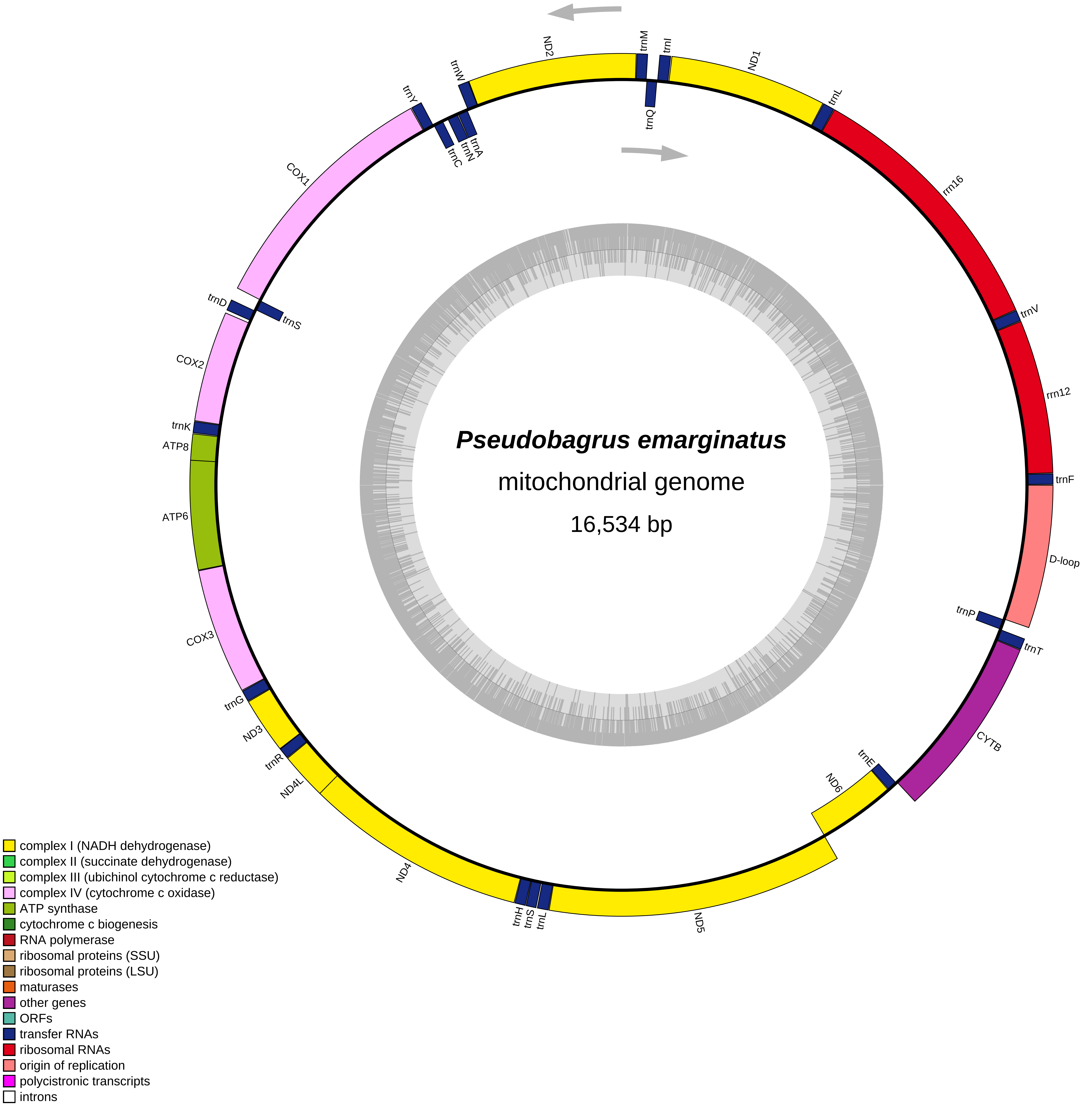

Pseudobagrus emarginatus
mitochondrial genome
16,534 bp
trnM
trnI
ND1
ND2
trnW
trnY
trnQ
trnA
trnN
trnC
trnL
rrn16
COX1
trnV
trnD
trnS
rrn12
COX2
trnK
ATP8
trnF
ATP6
D-loop
trnP
trnT
COX3
trnG
ND3
CYTB
trnE
trnR
ND6
ND4L
ND4
trnH
trnS
trnL
ND5
complex I (NADH dehydrogenase)
complex II (succinate dehydrogenase)
complex III (ubichinol cytochrome c reductase)
complex IV (cytochrome c oxidase)
ATP synthase
cytochrome c biogenesis
RNA polymerase
ribosomal proteins (SSU)
ribosomal proteins (LSU)
maturases
other genes
ORFs
transfer RNAs
ribosomal RNAs
origin of replication
polycistronic transcripts
introns

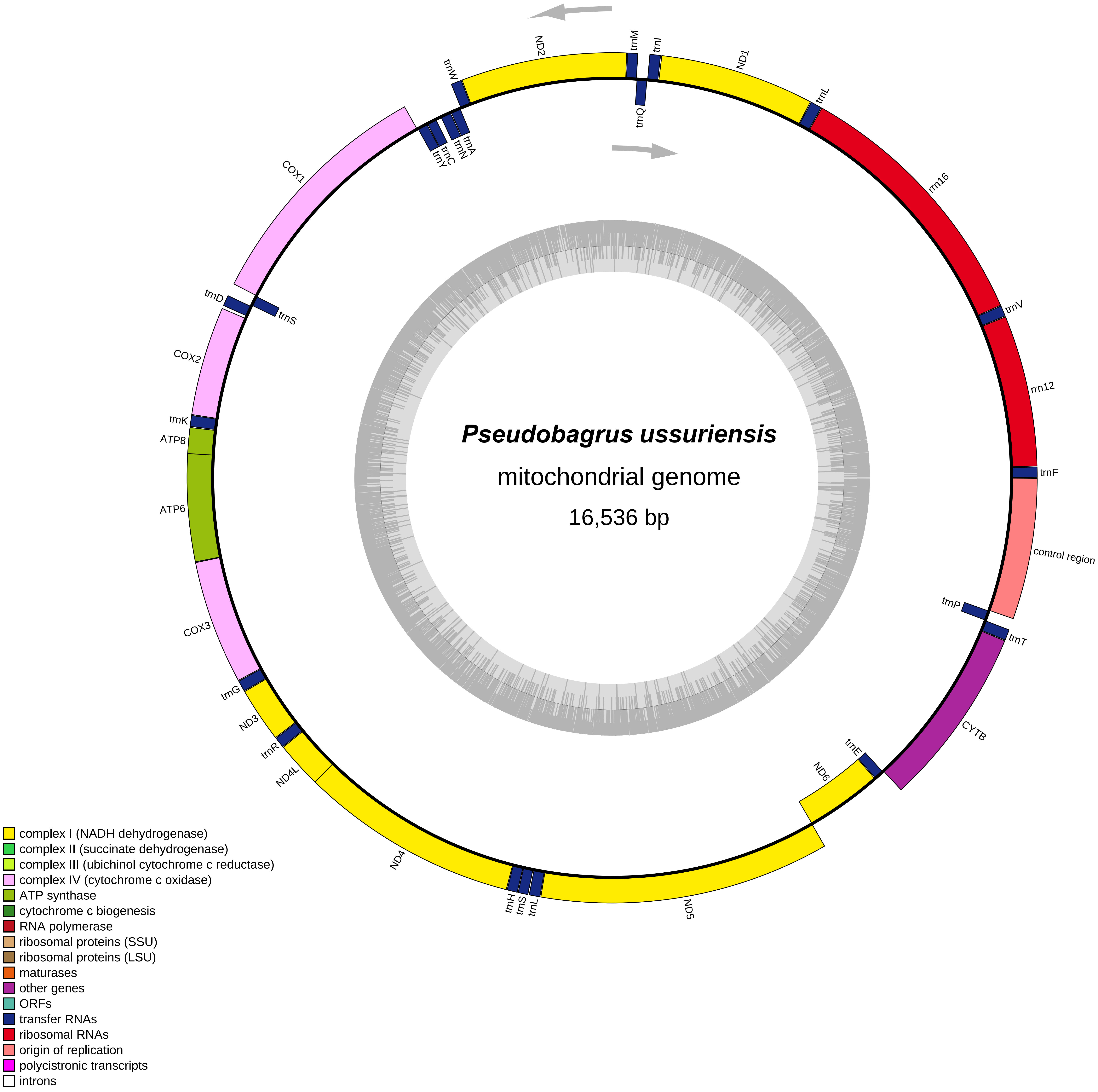
Pseudobagrus ussuriensis
mitochondrial genome
16,536 bp
trnM
trnI
ND2
ND1
trnW
trnQ
trnL
trnA
trnN
trnC
trnY
COX1
rrn16
trnD
trnS
trnV
COX2
rrn12
trnK
ATP8
trnF
ATP6
control region
trnP
trnT
COX3
trnG
CYTB
ND3
trnE
trnR
ND6
ND4L
ND4
trnH
trnS
trnL
ND5
complex I (NADH dehydrogenase)
complex II (succinate dehydrogenase)
complex III (ubichinol cytochrome c reductase)
complex IV (cytochrome c oxidase)
ATP synthase
cytochrome c biogenesis
RNA polymerase
ribosomal proteins (SSU)
ribosomal proteins (LSU)
maturases
other genes
ORFs
transfer RNAs
ribosomal RNAs
origin of replication
polycistronic transcripts
introns
