# Supplementary material for: Identified Optimal Codons and Phylogenetic Relationship in Pseudobagrus Species Based on Complete Mitogenomes
Source: Animals (Basel). 2026 Jan 16;16(2):279. doi: 10.3390/ani16020279 (PMC12837665; doi:10.3390/ani16020279)
Supplement: Supplementary file 1 [file animals-16-00279-s001.zip › Figure S2.pdf]

Pseudobagrus albomarginatus

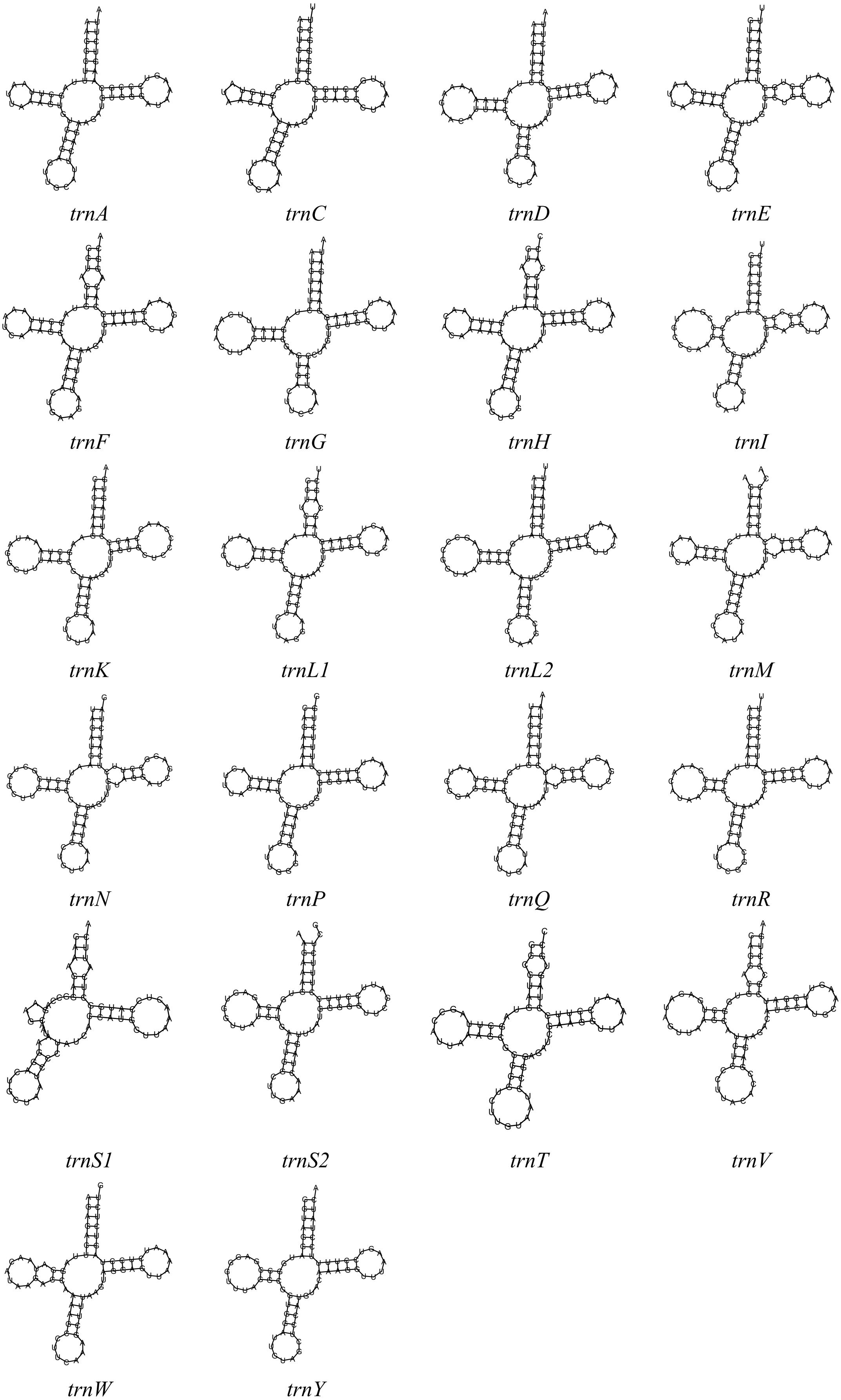

Pseudobagrus brachyrhabdion

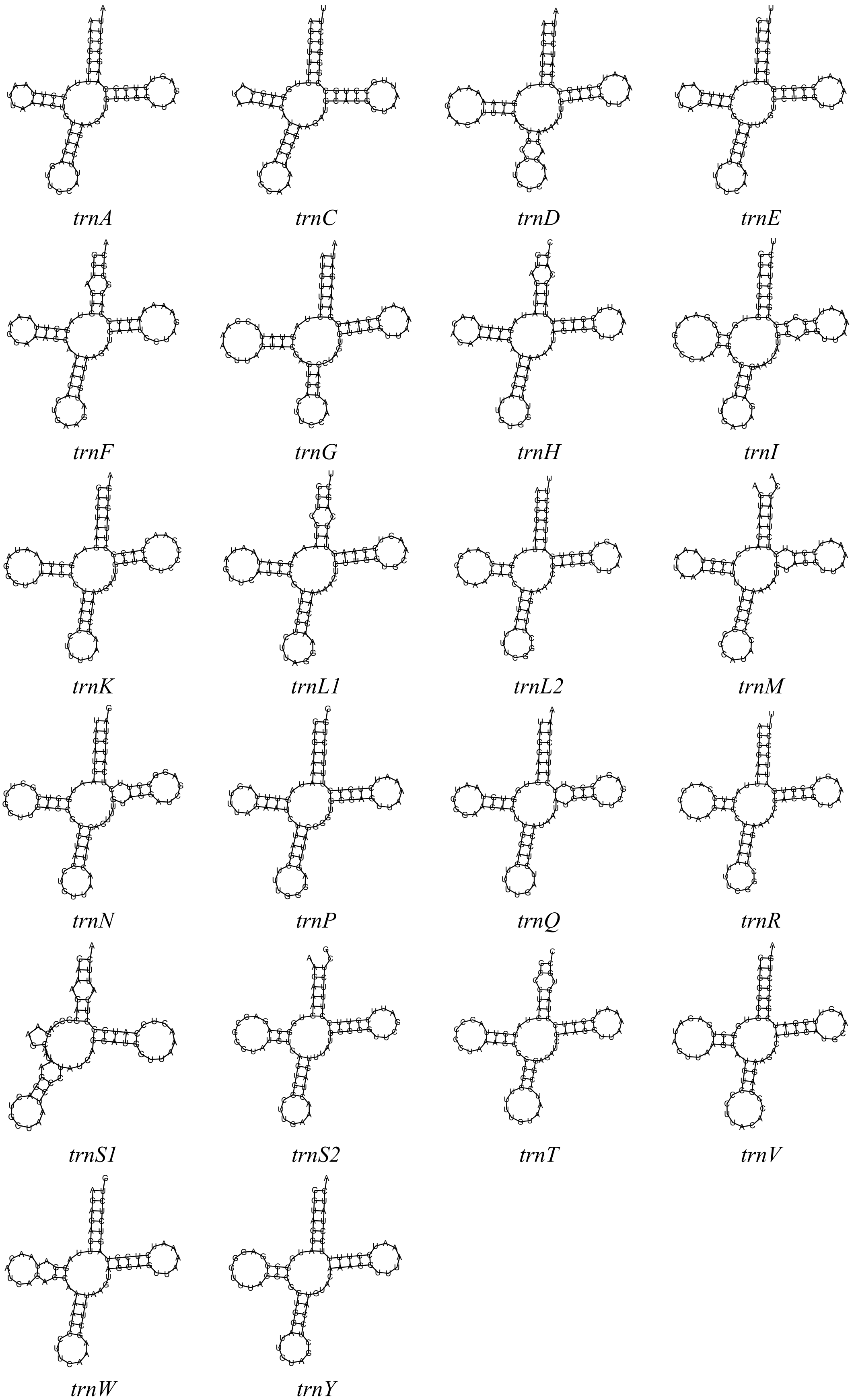

Pseudobagrus brevicaudatus

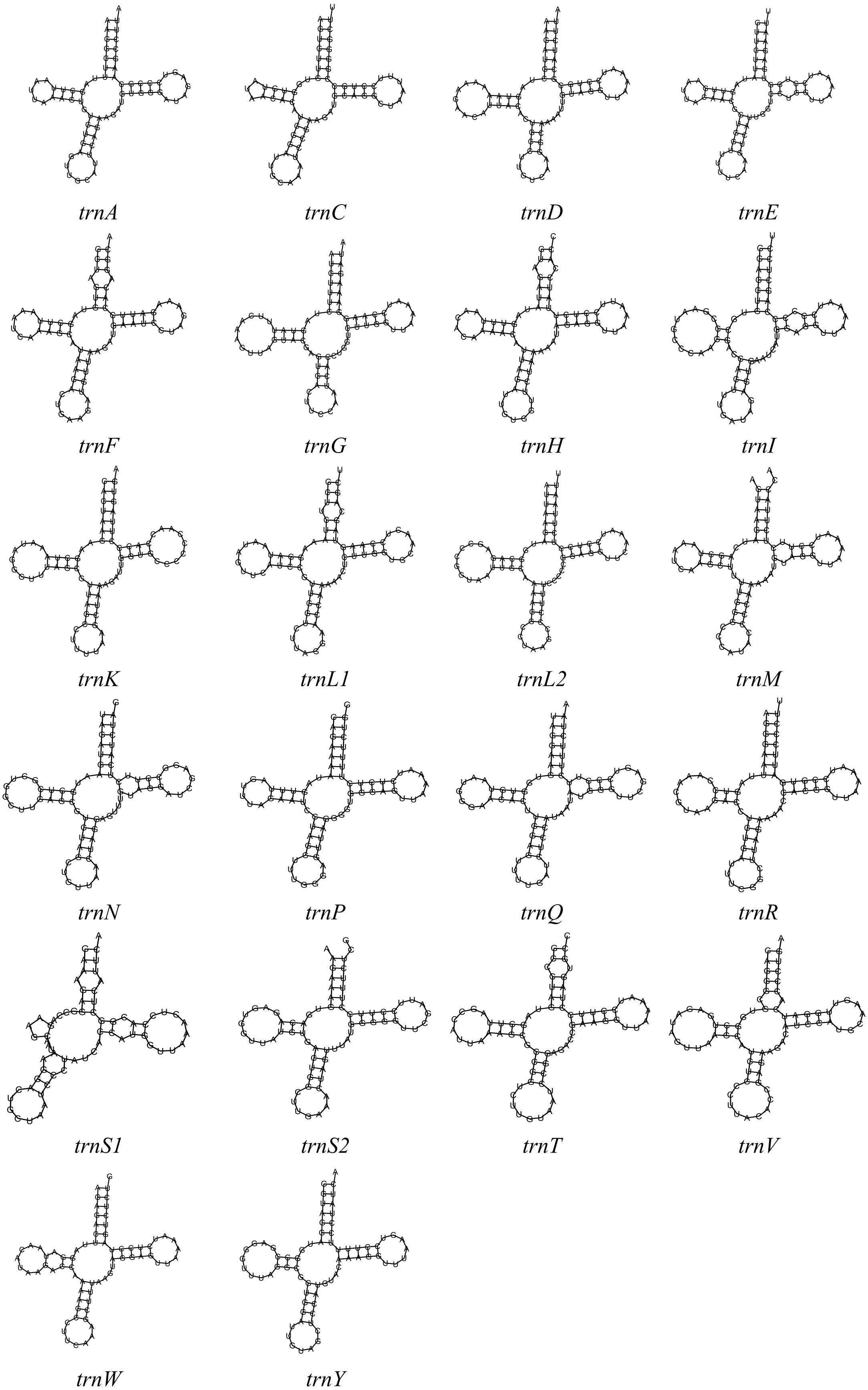

Pseudobagrus brevicorpus

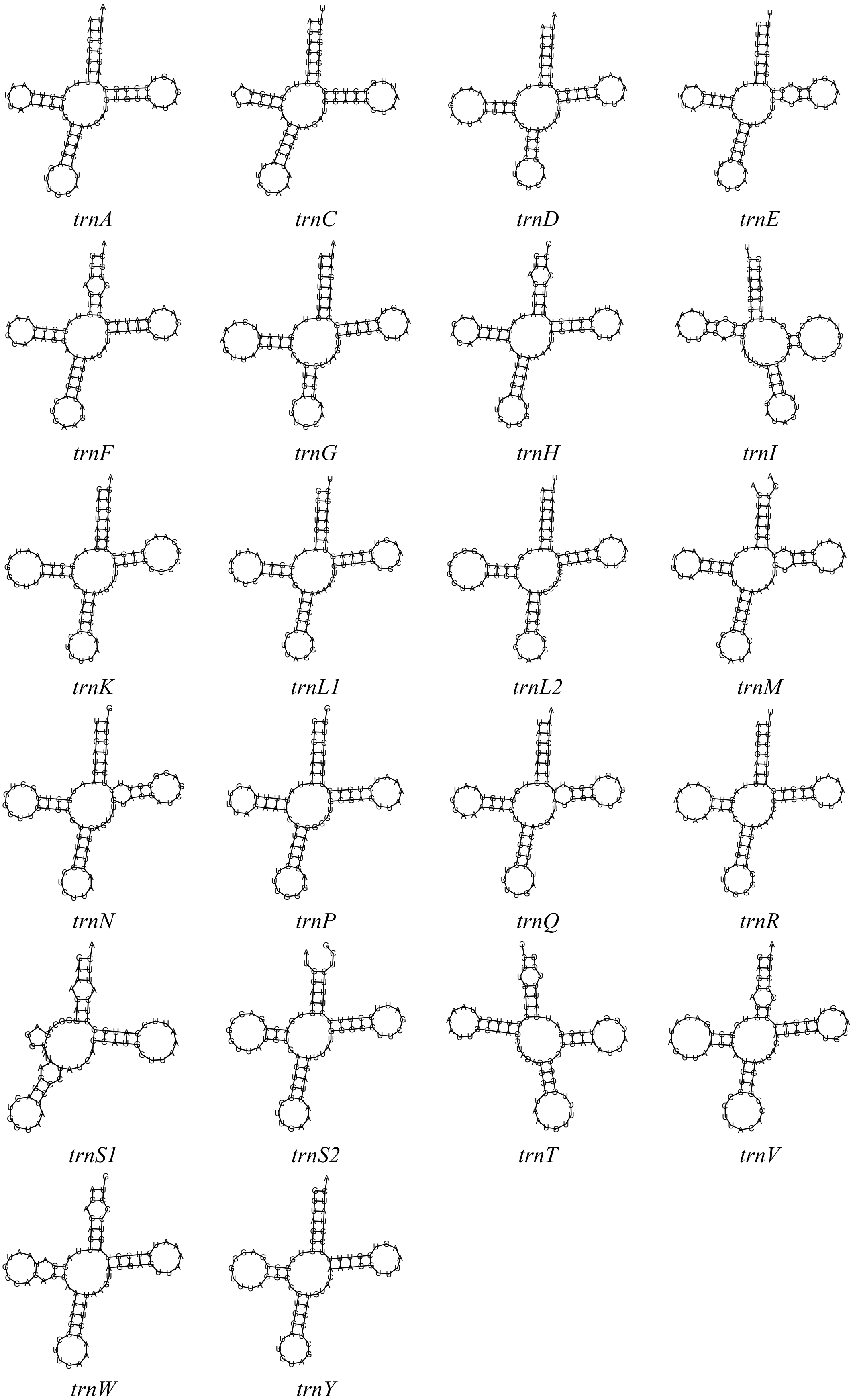

Pseudobagrus emarginatus

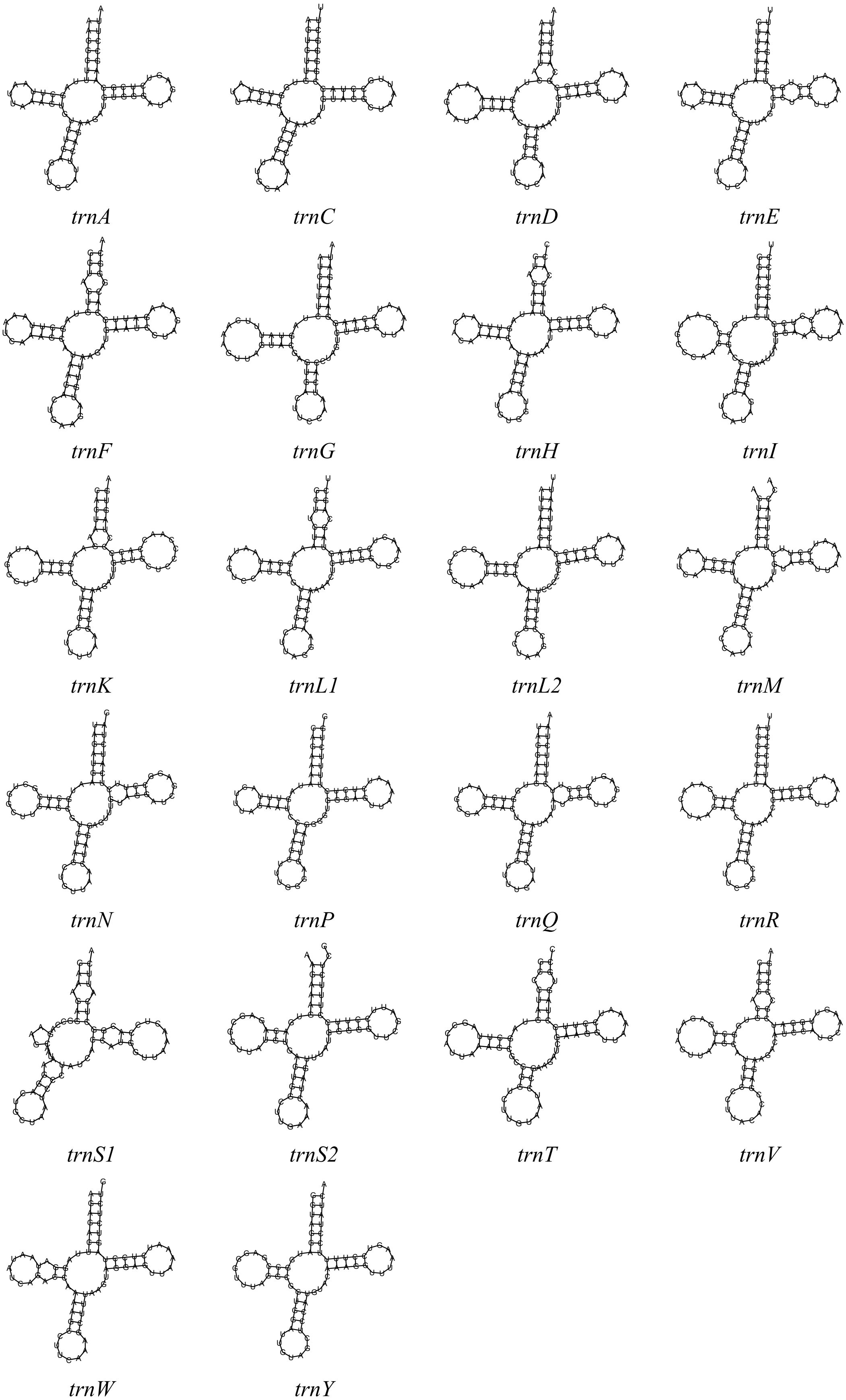

Pseudobagrus gracilis

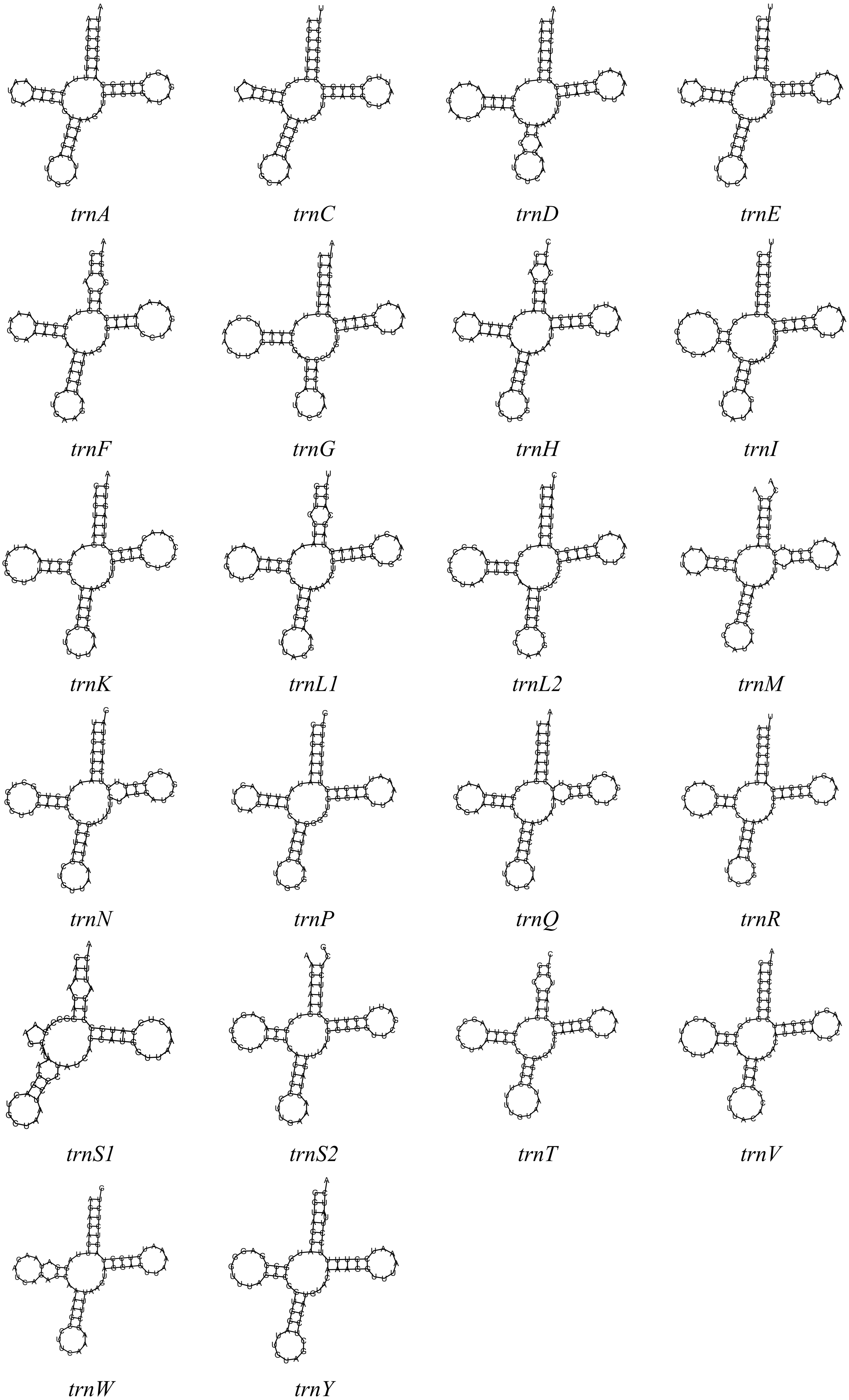

Pseudobagrus koreanus

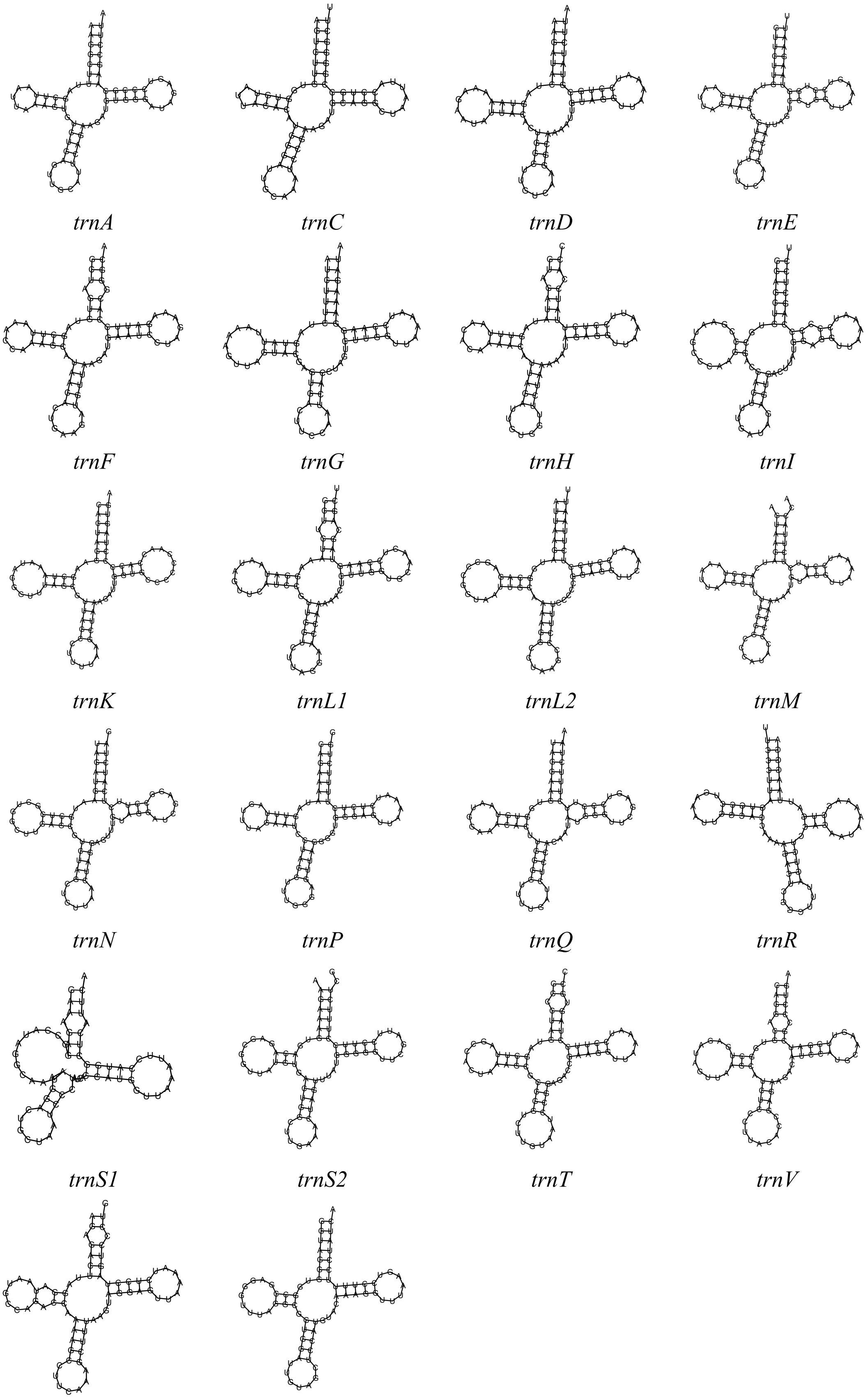

Pseudobagrus medianalis

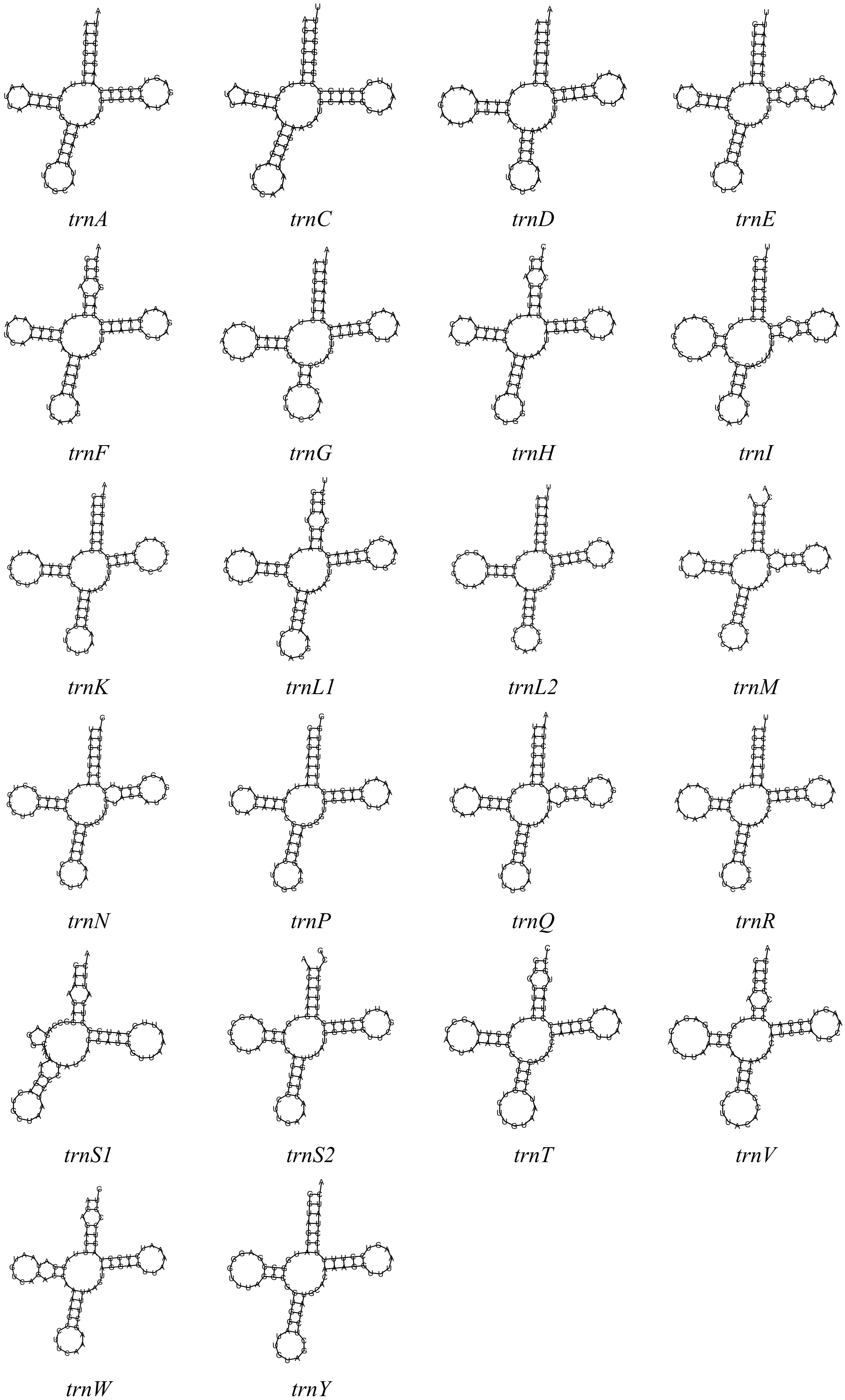

Pseudobagrus ondon

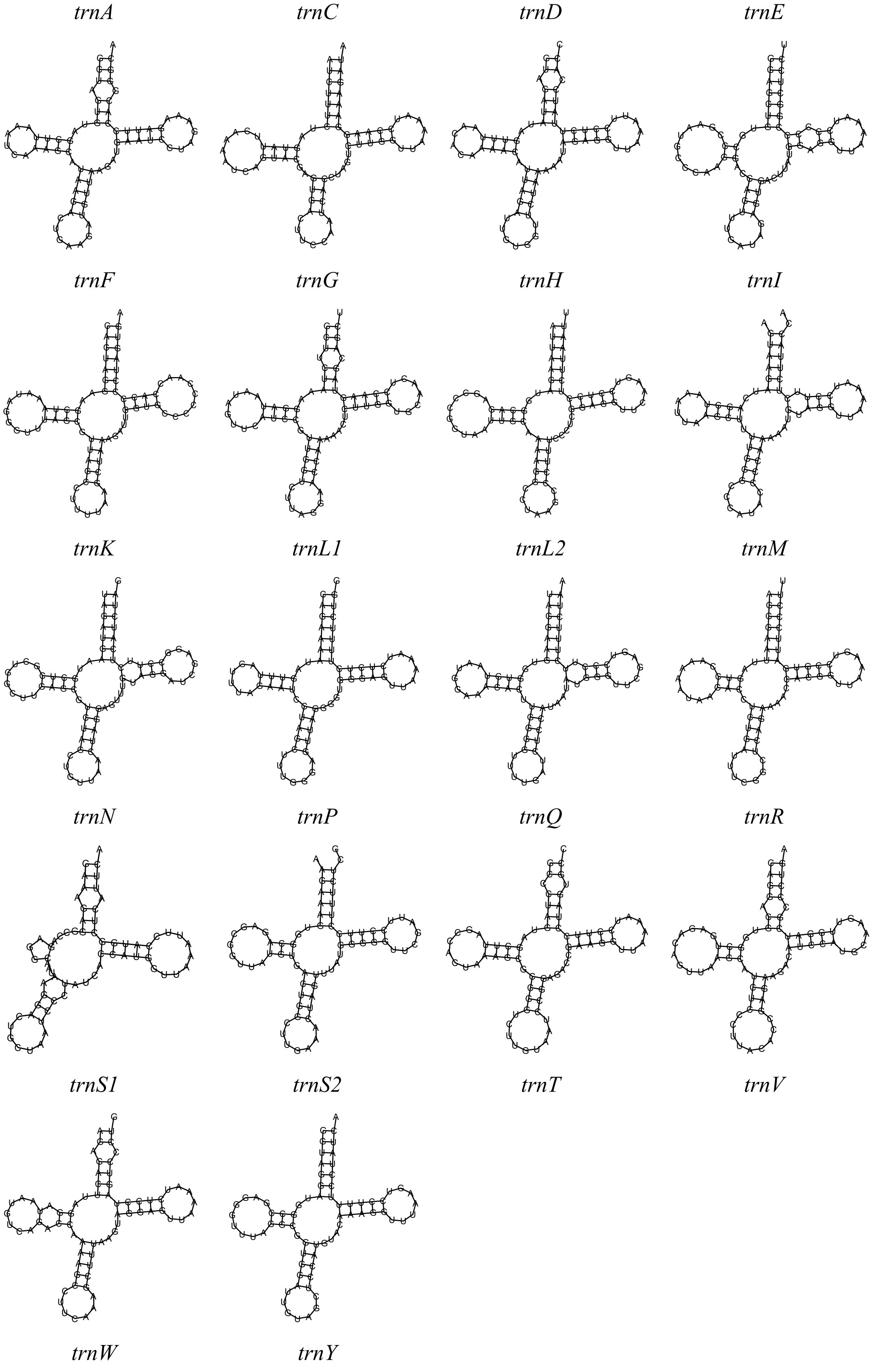

Pseudobagrus pratti

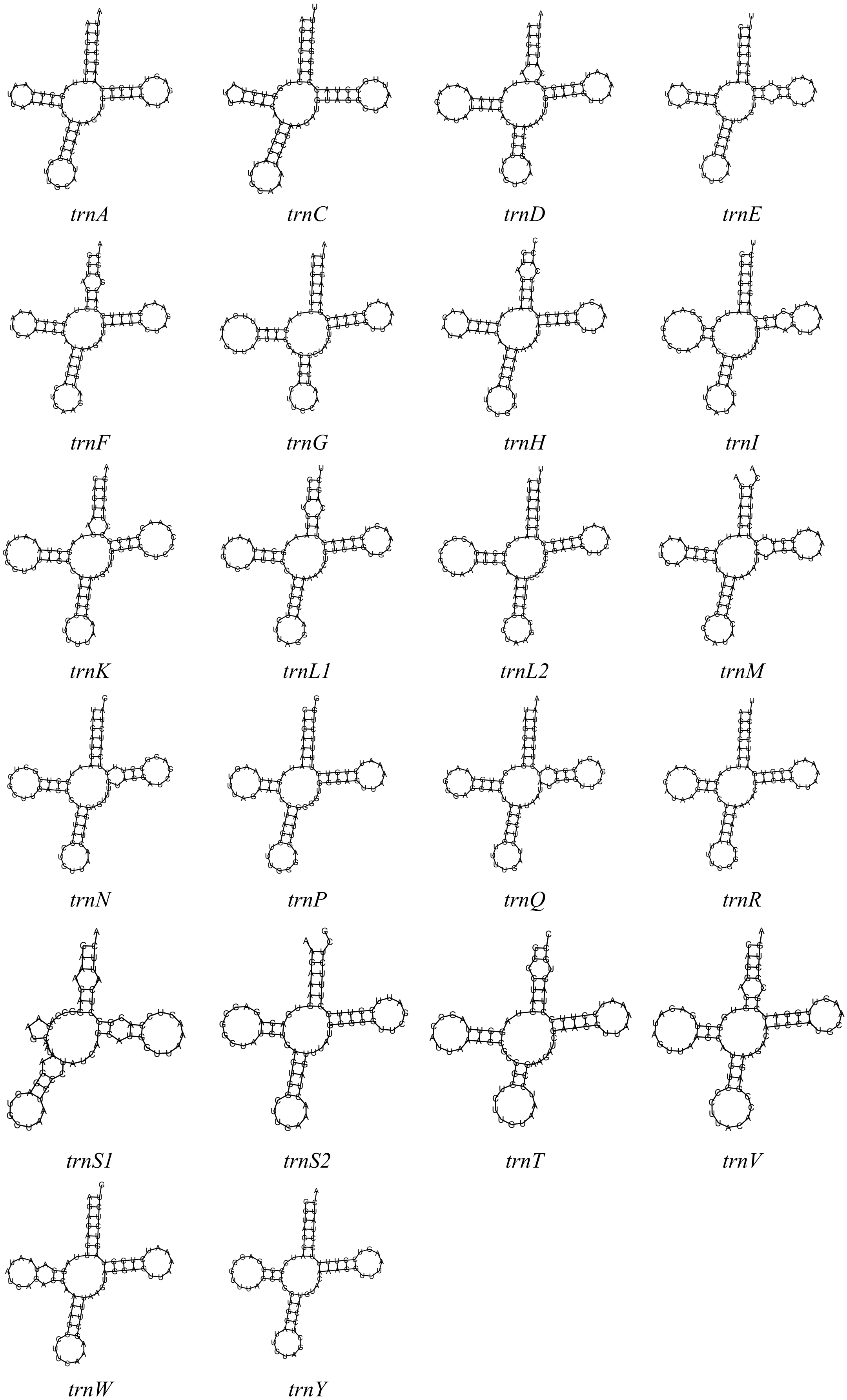

Pseudobagrus tenuis

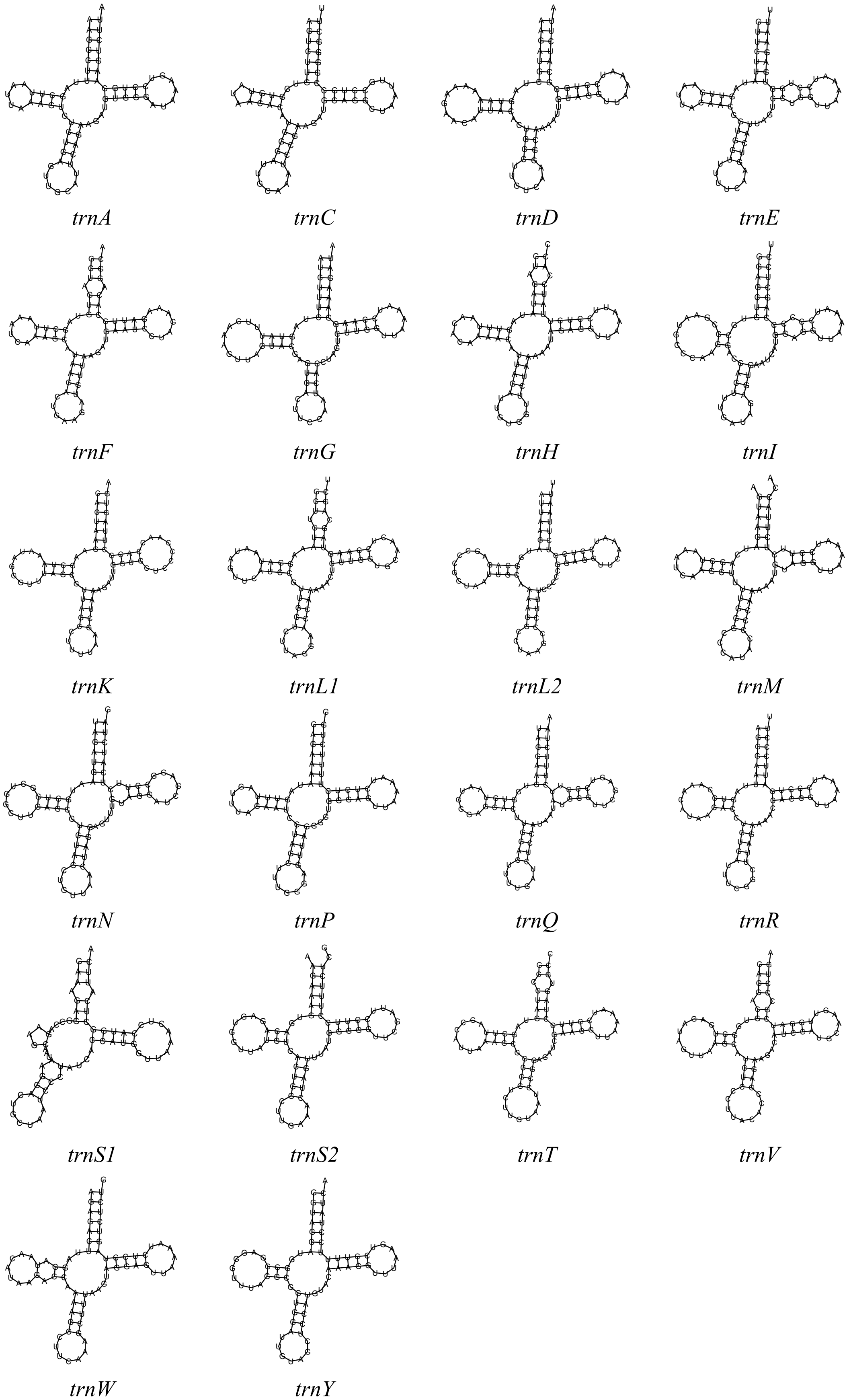

Pseudobagrus tokiensis

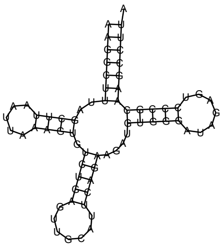

trnA

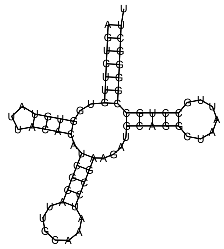

trnC

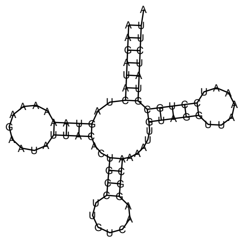

trnD

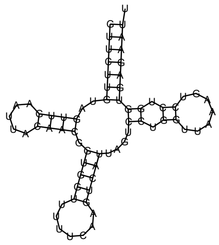

trnE

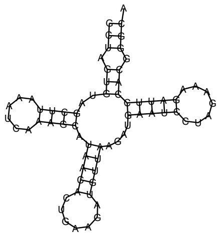

trnF

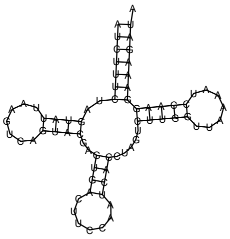

trnG

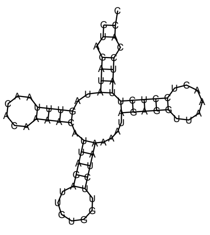

trnH

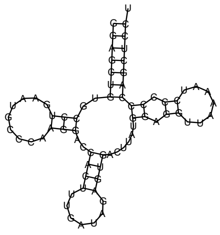

trnI

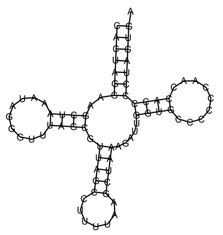

trnK

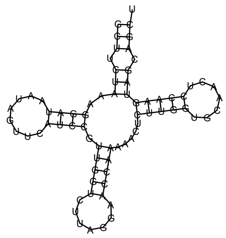

trnL1

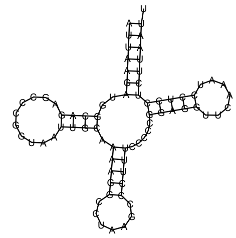

trnL2

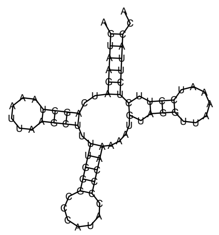

trnM

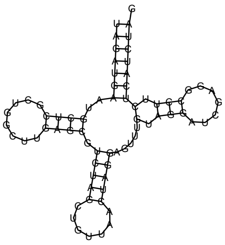

trnN

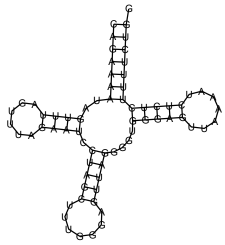

trnP

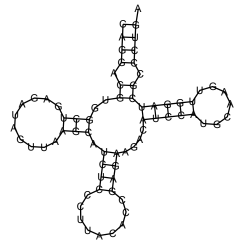

trnQ

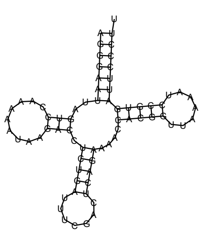

trnR

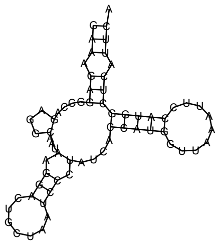

trnS1

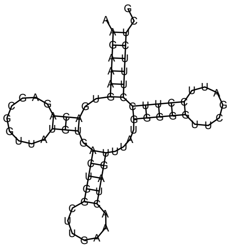

trnS2

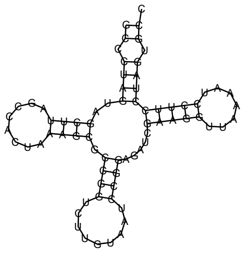

trnT

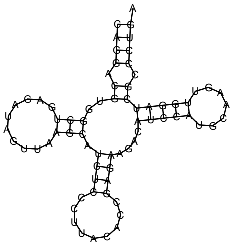

trnV

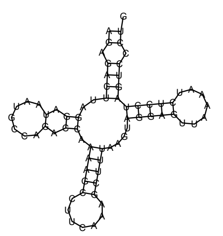

trnW

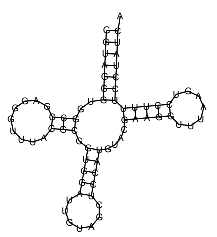

trnY

Pseudobagrus trilineatus

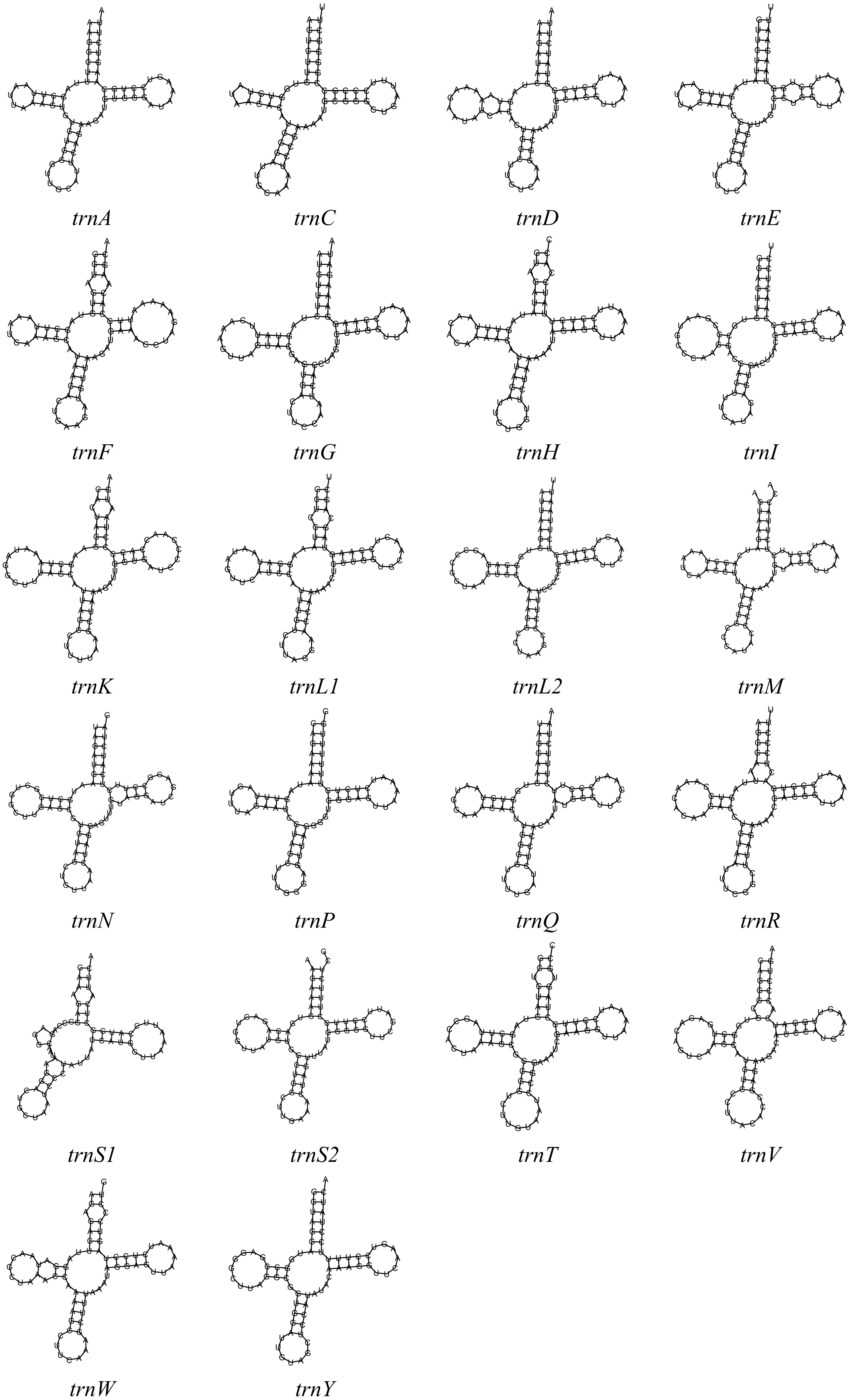

Pseudobagrus truncatus

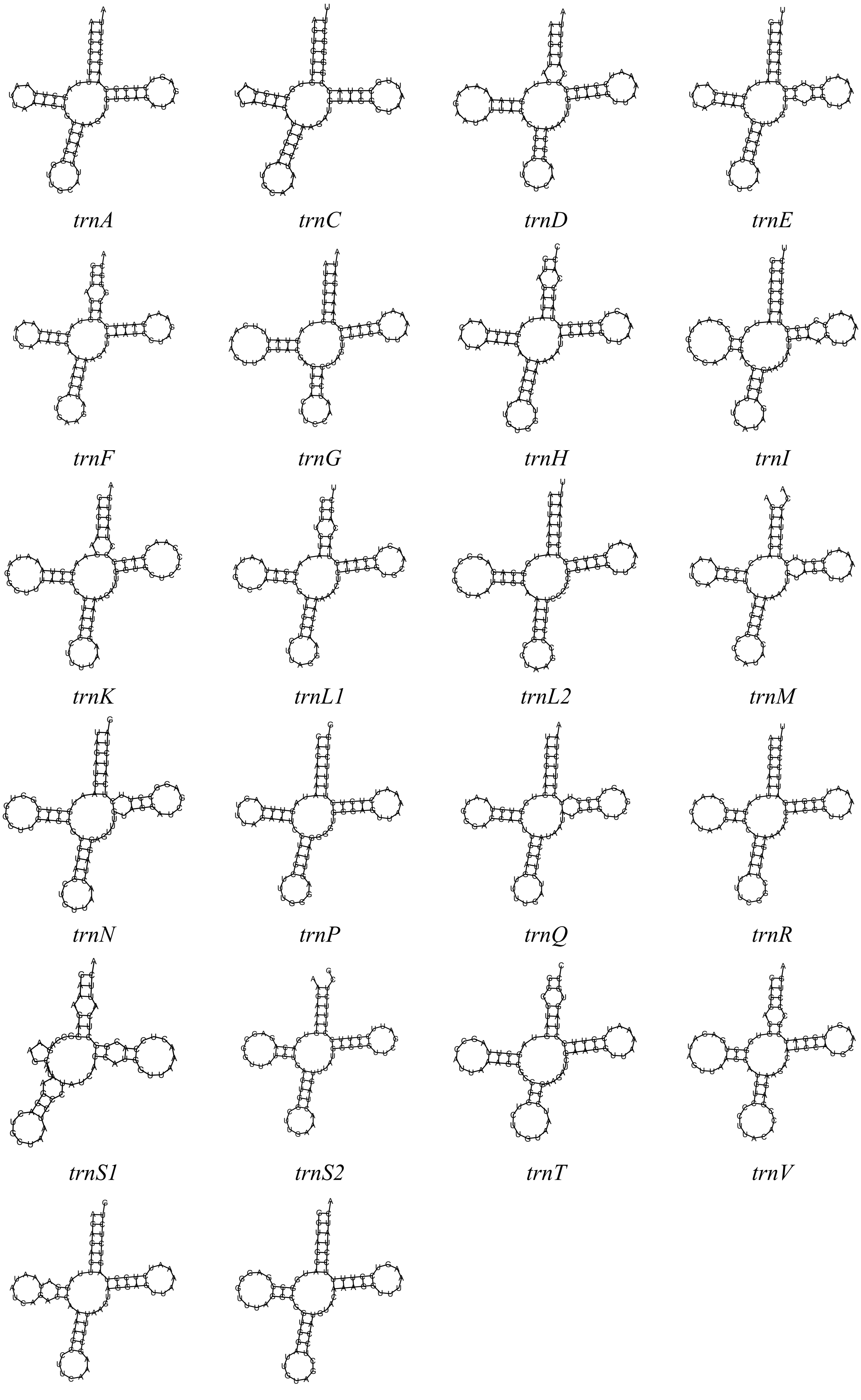

Pseudobagrus ussuriensis

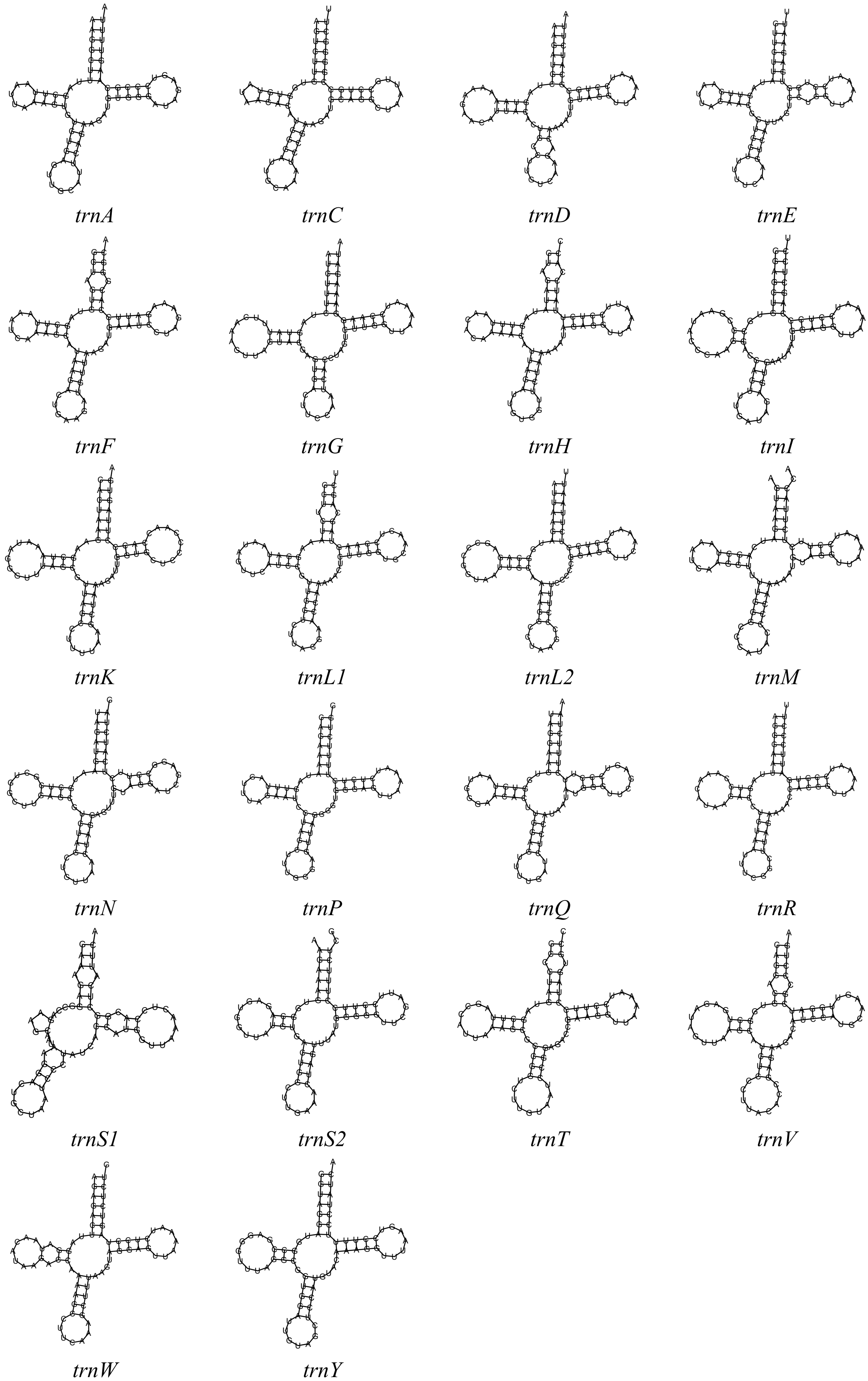

Figure S2 Schematic diagram of tRNAs secondary structures in the *Pseudobagrus* mitogenomes.
